# Identification of PRRG1 as a possible molecular target of pancreatic cancer

**DOI:** 10.1038/s41419-026-08832-9

**Published:** 2026-05-10

**Authors:** Jia-Jie Chen, Xiao-Ren Zhu, Qian-Hui Gu, Yuan-Yuan Liu, Min-Bin Chen

**Affiliations:** 1 Department of Radiotherapy and Oncology, Affiliated Kunshan Hospital of Jiangsu University, Kunshan, China; 2 Department of Radiotherapy and Oncology, Suzhou Medical College of Soochow University, The First People’s Hospital of Kunshan, Kunshan, China; 3 Clinical Research and Lab Center, Affiliated Kunshan Hospital of Jiangsu University, Kunshan, China; 4 Stem Cell Institute, Department of Development and Regeneration, Katholieke Universiteit (KU) Leuven, Leuven, Belgium

**Keywords:** Tumour biomarkers, Cancer microenvironment, Pancreatic cancer, Oncogenes

## Abstract

In this study, we investigated the expression patterns, biological functions, and molecular mechanisms of Proline-rich γ-carboxylated Gla protein 1 (PRRG1) in pancreatic cancer pathogenesis. Our bioinformatics analysis revealed that PRRG1 expression is markedly upregulated in human PC tissues compared to normal pancreatic tissues, with elevated levels significantly correlating with poor prognosis and advanced histological grade. We verified the high expression of PRRG1 in pancreatic cancer tissue specimens and pancreatic cancer cell lines. Using established PC cell lines (CFPAC-1 and PATU-8988T), we demonstrated that shRNA-mediated PRRG1 silencing effectively suppressed malignant phenotypes, including cell viability, proliferation, migration, and invasion in vitro. Conversely, lentivirus-induced PRRG1 overexpression enhanced these oncogenic behaviors. RNA-sequencing analysis identified the PI3K-Akt signaling pathway as a key downstream effector of PRRG1, with pathway activation status directly correlating with PRRG1 expression levels. Mechanistically, we identified KLF4 as a critical transcription factor binding to the PRRG1 promoter region. In vivo, PRRG1 knockdown inhibited tumor growth and PI3K-Akt activation in subcutaneous xenograft models, while PRRG1 overexpression accelerated tumor progression. Low-dose warfarin (2uM) decreased the levels of PRRG1 and GAS6/AXL axis, markedly suppressed the pro-tumorigenic effects driven by PRRG1 overexpression in vitro and in vivo. Notably, single-cell sequencing analysis revealing high PRRG1 expression specifically in PC epithelial cells. These PRRG1-positive epithelial cells not only exhibited enriched PI3K-Akt signaling activity but also showed significant interactions with macrophages and endothelial cells, which were further validated in immunocompetent models in vivo. However, warfarin effectively reversed the PRRG1 overexpression–driven changes in TME. In conclusion, our findings establish PRRG1 as a key driver of pancreatic cancer progression through PI3K/Akt pathway activation and KLF4-mediated transcriptional regulation. PRRG1 facilitates the establishment of a pro-tumorigenic and immunosuppressive TME in PC. Low-dose warfarin significantly suppressed the pro-tumorigenic effects and the PRRG1 overexpression–driven alterations in the tumor immune microenvironment.

## Introduction

Pancreatic cancer (PC) is among the most aggressive and lethal human malignancies. Recent data indicate that the 5-year survival rate for pancreatic ductal adenocarcinoma (PDAC) remains dismal, at approximately 13% [1]. This poor prognosis is largely attributable to late-stage diagnosis in most patients, highlighting the critical need for improved early detection methods [2, 3]. Current non-surgical treatment modalities, including chemotherapy [4], radiotherapy [5], targeted therapy [6], immunotherapy [7] are significantly limited by challenges, such as drug resistance and tumor immune evasion mechanisms. These clinical hurdles underscore the urgent necessity to identify novel molecular targets and develop more effective therapeutic strategies for this devastating disease.

Vitamin K-dependent (VKD) proteins have emerged as crucial mediators of cancer progression [8–11]. These proteins undergo a unique γ-carboxylation modification catalyzed by vitamin K-dependent γ-glutamyl carboxylase [12], which converts specific glutamic acid (Glu) residues to γ-carboxylated glutamic acid (Gla) [13]. This post-translational modification is essential for VKD protein activation and enables their participation in various physiological and pathological processes, including cancer development [14].

Among VKD proteins, Protein S [15] and Gas6 [16] serve as shared ligands for TAM receptor tyrosine kinases (TYRO3, AXL, and MERTK), which are known to drive multiple oncogenic processes [17, 18]. Emerging evidence demonstrates that the Protein S/TYRO3 axis promotes tumor cell migration, proliferation, and angiogenesis in lung cancer [8]. Furthermore, Protein S has been shown to activate the TYRO3/ERK signaling pathway, thereby suppressing cancer cell apoptosis and facilitating tumor progression [9]. Similarly, the Gas6/AXL axis plays a pivotal role in regulating tumor cell growth, metastasis, and epithelial-mesenchymal transition (EMT) [10]. Warfarin, an oral coumarin anticoagulant, leading to a reduction in vitamin K hydroquinone (KH) and subsequent inhibition of γ-glutamyl carboxylation [19, 20]. This prevents VKD proteins from forming Gla residues and thereby impairs their biological functions. Notably, low-dose warfarin (2uM) has been shown to suppress PC progression by inhibiting the GAS6/AXL axis [11], highlighting the therapeutic potential of targeting VKD protein–related signaling pathways.

The proline-rich Gla (PRRG) protein family, comprising PRRG1, PRRG2, PRRG3 and PRRG4, represents a unique class of vitamin K-dependent single-pass transmembrane proteins [21–23]. These proteins are structurally characterized by an extracellular γ-carboxylated glutamic acid (Gla) domain and distinctive intracellular motifs [22, 24]. Emerging evidence implicates PRRG family members as important regulators in cancer pathogenesis. PRRG4 recruits E3 ubiquitin ligase NEDD4 through its LPSY and PPPY motif, and induces the ubiquitination and degradation of cancer suppressor Robo1, which promoting the activation of Src and FAK and breast cancer metastasis [25]. Additionally, PRRG4 enhances cell migration via the Src-STAT3-POLG signaling axis in breast cancer models [26]. PRRG1 has been associated with poor prognosis in various malignancies including glioma [27], hepatocellular carcinoma [28], and breast cancer [29]. Wu et al. [30] suggested that PRRG1 promotes PDAC progression through stabilizing KRAS and EGFR. PRRG1 appears to be a novel oncogenic gene. However, more information about precise mechanisms of PRRG1 in PC remain to be elucidated, and further studies are required to evaluate its potential as a therapeutic target for this malignancy.

## Materials and Methods

### Reagents and antibodies

Cell Counting Kit-8 (CCK-8) was purchased from Dojindo Co. (Kumamoto, Japan). LY294002, DAPI (4’,6-diamidino-2-phenylindole) and EdU (5-ethynyl-20-deoxyuridine), were provided by Thermo-Fisher Invitrogen (Shanghai, China). Antibiotics, puromycin and medium were purchased from Sigma-Aldrich Chemicals (St. Louis, Mo). RNA reagents and other transfection reagents were obtained from Thermo-Fisher Invitrogen (Carlsbad, CA). Warfarin and Vitamin K were purchased from MedChemExpress (MCE, Monmouth Junction, NJ, USA). The primary antibodies used were following: anti-PRRG1 (for Western blotting) (14103-1-AP, Proteintech, 1:1000), anti-PRRG1 (for Immunohistochemistry) (orb312475, Biorbyt, 1:1000), anti-PRRG4 (for Western blotting) (PA5-115801, Thermo, 1:1000), Tubulin (ab179513, Abcam, 1:5000), anti-mTOR (#2983, Cell Signaling Technology, 1:2000), anti-phospho-mTOR (ab109268, Abcam, 1:2000), anti-Akt1/2/3 (BM4400, BOSTER, 1:2000), anti-phospho-Akt (Ser-473) (#4060, Cell Signaling Technology, 1:2000), anti-S6 (#2217, Cell Signaling Technology, 1:3000), anti-phospho-S6 (#4858, Cell Signaling Technology, 1:3000), anti-4E-BP1 (#9644, Cell Signaling Technology, 1:3000), anti-phospho-4E-BP1 (#9456, Cell Signaling Technology, 1:3000), anti-Gas6 (13795-1-AP, Proteintech, 1:500), anti-AXL (13196-1-AP, Proteintech, 1:3000), anti-phospho- AXL(Tyr702) (AF8523, Affinity, 1:3000), anti-PRRG1 (for mIHC) (DF15899, Affinity, 1:50), anti-Ki67 (ab16667, Abcam, 1:200), anti-PANCK (ab7753, Abcam, 1:200), anti-Ly6C (ab314120, Abcam, 1:100), anti-F4/80 (ab111101, Abcam, 1:50), anti-CD31 (ab182981, Abcam, 1:2000).

### Bioinformatics analysis

Pan-Cancer data was from the XENA database that has been uniformly processed by the Toil process (UCSC Xena (xenabrowser.net)). The Cancer Genome Atlas (TCGA) database and the Genotype-Tissue Expression (GTEx) project were consulted to analyze PRRG1 and KLF4 expression in PC tissues and corresponding normal tissues. Further clinical bioinformatic analysis based on TCGA database was employed to study whether high PRRG1 expression associated with poor prognosis, including Kaplan-Meier survival analysis, ROC curve analysis and clinical subgroup analysis. Additionally, KEGG enrichment analysis was employed to analyze signaling pathways that PRRG1-associated DEGs enriched in. Single-cell sequencing analysis used the scRNA-seq data from dataset GSE155698 of the GEO database.

### Cell culture and tissue microarray

Four established human PC cell lines, CFPAC-1, PATU-8988T, MIA PaCa-2 and PANC-1, and normal pancreas cells (HPNE) were purchased from Institute of Biochemistry and Cell Biology, Chinese Academy of Sciences (Shanghai, China). Mouse pancreatic ductal adenocarcinoma (PDAC) cell line (PANC02) was purchased from Haixing Biotechnology Co (Suzhou, China). All cell lines were authenticated using short tandem repeat (STR) analysis performed by a commercial service provider to confirm the identity of each cell line. In addition, all cell lines were routinely tested and confirmed to be free of mycoplasma contamination. Cells were cultivated in Dulbecco’s Modified Eagle Medium (DMEM) medium (Gibco, Waltham, MA, USA) with 10% fetal bovine serum (FBS, Gibco, Waltham, MA) and 1% penicillin/streptomycin (Gibco, Waltham, MA). The medium was refreshed every two days. The human PC tissue microarray (No. HPanA170Su05) was procured and analyzed by Shanghai SuperChip Co (Shanghai, China).

### Quantitative real-time PCR (qPCR)

Total RNA was extracted from cells or tissues using the RNA-Quick Purification Kit (ES Science) according to the manufacturer’s protocol. RNA quality was assessed by NanoDrop 2000 (Thermo Fisher Scientific) and Agilent 2100 Bioanalyzer, and only samples with A260 / A280 ratios of 1.8–2.0 or RIN ≥ 7 were used. Genomic DNA was removed using RNase-free gDNase (CWBIO).

cDNA was synthesized from 0.5 to 1 µg of total RNA using the FastKing RT Kit (CWBIO) according to the manufacturer’s instructions. Quantitative PCR was performed using a SYBR Green detection system. Each 20 µL reaction contained 10 µL of 2× UltraSYBR Mixture (CWBIO), 0.4 µL of forward primer (10 µM), 0.4 µL of reverse primer (10 µM), 0.8 µL of cDNA template, and nuclease-free water to a final volume of 20 µL. Amplification and detection were conducted using the following thermal cycling conditions: 95 °C for 10 min, followed by 40 cycles of 95 °C for 15 s and 60 °C for 60 s. A melting curve analysis (60 °C–95 °C, 0.5 °C increment per step) was performed to confirm amplification specificity and the absence of primer dimers.

Relative gene expression levels were calculated using the 2^-ΔΔCt method [31]. GAPDH was used as an internal control. Amplification efficiency was validated for each primer pair using a 10-fold serial dilution standard curve, and only primers with efficiency values between 90 - 110% and R² ≥ 0.99 were included. Efficiency validation followed the MIQE guidelines [32]. Primer sequences are provided in Supplementary Table (Table S1). All reactions were performed in technical triplicates.

### Western blotting

Cells or tissues were lysed in RIPA buffer supplemented with protease and phosphatase inhibitors (Roche). Protein concentrations were determined using the BCA protein assay (Thermo Fisher). Equal amounts of protein were separated by SDS-PAGE (Bio-Rad, 100 V, 90 min) and transferred to PVDF membranes (0.45 μm pore size, Millipore). Membranes were blocked with 5% non-fat milk in TBST, and then incubated overnight at 4 °C with primary antibodies. After washing, membranes were incubated with HRP-conjugated secondary antibodies for 1 h at room temperature. Protein bands were visualized using enhanced chemiluminescence (ECL) detection reagents. Band intensities were quantified using ImageJ software (NIH, USA), normalized to β-Tubulin, and expressed relative to control samples.

### CCK-8 cell viability assay

Cell viability was evaluated using the Cell Counting Kit-8 (Dojindo, Japan) according to the manufacturer’s instructions. Briefly, cells were seeded into 96-well plates at a density of (2–3) × 10³ cells/well in 100 µL of complete medium and cultured for 96 h. After the indicated treatments, 10 µL of CCK-8 reagent was added to each well and incubated for 1–4 h at 37 °C. The absorbance was measured at 450 nm using a microplate reader (Bio-Rad). The relative cell viability was calculated as the percentage of the control group.

### EdU /DAPI double staining assay

Cell proliferation was assessed using the 5-ethynyl-2′-deoxyuridine (EdU) assay with a commercial kit provided by Thermo-Fisher Invitrogen (Shanghai, China), following the manufacturer’s protocol. Briefly, cells were seeded in 24-well plates or chamber slides and cultured for 72 h. After the indicated treatments, cells were incubated with EdU working solution (50 μM) for 2 h at 37 °C.

Subsequently, cells were fixed with 4% paraformaldehyde for 15 min, permeabilized with 0.3% Triton X-100 for 10 min, and stained with the Apollo reaction cocktail to label the incorporated EdU. Cell nuclei were counterstained with Hoechst 33342 for 10 min. Images were captured using a fluorescence microscope (Olympus IX73), and the percentage of EdU-positive cells was quantified using ImageJ software.

### Colony formation assay

The clonogenic ability of cells was assessed using a colony formation assay. Briefly, cells were seeded into 6-well plates at a density of 500–1000 cells per well and cultured for 14 days until visible colonies formed. The medium was replaced every 2–3 days. At the end of the incubation, colonies were washed twice with PBS, fixed with 4% paraformaldehyde for 15 min, and stained with 0.1% crystal violet for 15–30 min at room temperature. The plates were rinsed gently with water and air-dried. Colonies containing more than 50 cells were counted under a microscope.

### Migration assay

For the migration assay, 5 × 10⁴–1 × 10⁵ cells in serum-free medium were seeded into the upper chambers. The lower chambers were filled with medium containing 10% fetal bovine serum (FBS) as a chemoattractant. After 24 h incubation at 37 °C, non-migrated cells on the upper surface of the membrane were gently removed with a cotton swab. The migrated cells on the lower surface were fixed with 4% paraformaldehyde for 15 min, stained with 0.1% crystal violet for 15–30 min, and counted under a microscope in at least five random fields.

### Invasion assay

For the invasion assay, the upper chambers were pre-coated with Matrigel (BD Biosciences, USA) diluted 1:8 in serum-free medium and allowed to solidify at 37 °C for 1–2 h. Then, 5 × 10⁴–1 × 10⁵ cells suspended in serum-free medium were added to the upper chamber, and the lower chamber was filled with medium containing 10% FBS. After 2 h incubation, the invaded cells were fixed, stained, and counted as described for the migration assay.

### PRRG1 shRNA

Three different lentiviral shRNAs targeting non-overlapping sequences of human PRRG1 (“shPRRG1-a/-b/-c”) as well as the scramble control (“shC”) designed and validated by Genechem Co (shanghai, China), were transfected into CFPAC-1 and PATU-8988T cells (seeded into six-well culture plates at 60–70% of confluence). Stable cells were established after two weeks selection by puromycin (2.0 µg/mL). Silencing of PRRG1 in stable cells was verified by qPCR and Western Blotting assays. The sequences of the shRNAs are provided in Supplementary Table (Table S2).

### PRRG1/Prrg1 overexpression

The lentiviral particles encoding PRRG1 (PRRG1 for human) cDNA (“OE-PRRG1”) and the empty vector (“Vector”), provided by Genechem (Shanghai, China), were transfected into CFPAC-1 and PATU-8988T cells. The lentiviral particles encoding Prrg1 (Prrg1 for mouse) cDNA (“OE-Prrg1”) and the empty vector (“Vector”), provided by Genechem (Shanghai, China), were transfected into PANC02 cells. Cells were seeded into six-well culture plates at 60–70% of confluence. Stable cells were established after selection by puromycin (2.0 µg/mL) for two weeks. PRRG1 overexpression was verified by qPCR and Western Blotting assays.

### RNA interference (RNAi)

CFPAC-1 cells were transfected with a small interference RNA (siRNA) targeting KLF4, EHF and PPARA as well as negative control siRNA by Lipofectamine 2000 (Invitrogen, Carlsbad, CA). After transfection for 48 h, cells were used for further experiments. The verified siRNAs used were purchased from Genechem (Shanghai, China). The sequences of the siRNAs are provided in Supplementary Table (Table S3).

### Immunohistochemistry (IHC)

Paraffin-embedded tissue sections were deparaffinized in xylene and rehydrated through a graded ethanol series. Antigen retrieval was performed by heating the sections in citrate buffer (pH 6.0). Endogenous peroxidase activity was blocked with 3% hydrogen peroxide, followed by blocking with 5% bovine serum albumin (BSA). Sections were incubated overnight at 4 °C with primary antibodies. After washing, the sections were incubated with HRP-conjugated secondary antibodies at room temperature for 1 h. Staining was visualized using a DAB substrate kit and counterstained with hematoxylin. Images were captured with a light microscope.

### Multiplex Immunohistochemistry (mIHC)

Paraffin-embedded tissue sections were deparaffinized in xylene and rehydrated through a graded ethanol series. Antigen retrieval was performed by heating the sections in citrate buffer (pH 6.0). Endogenous peroxidase activity was blocked with 3% hydrogen peroxide for 10–15 min, and non-specific binding was blocked with 5% bovine serum albumin (BSA) for 1 h.

Sections were incubated overnight at 4 °C with primary antibodies specific to the antigens of interest. After washing, secondary antibodies conjugated with different enzymes or fluorophores were applied for 1 h at room temperature. For enzyme-based detection, HRP or AP-conjugated secondary antibodies were used with appropriate chromogenic substrates. For fluorescence-based detection, secondary antibodies conjugated with fluorophores were used, and images were captured using a fluorescence or confocal microscope.

Finally, sections were counterstained with a nuclear stain to visualize tissue morphology. The expression patterns of multiple targets were analyzed based on the different colors or fluorescence intensities.

### The dual-luciferase reporter assay

The 293 T cells were cultured with DMEM medium containing 10% serum. Logarithmic 293 T cells were inoculated into 24-well plates at a density of 1×105 cells /mL. They were divided into 4 groups, pcDNA3.1 + PGL4.11-PRRG1-WT/MU, PCDNA3.1-KLF4 + PGL4.11-PRRG1-WT/MU, and each group had three replicates. After overnight cell culture, transfection mixture was prepared, which consisted of liquid A (50 μL OPTI-MEM + 0.6 μg target plasmid or unloaded plasmid +20pmol miRNA-mimics/NC), liquid B (50 μL OPTI-MEM + 2 μL Lipofectamine 2000), and liquid B (50 μL Opti-Mem +2 μL Lipofectamine 2000). Liquid A and B were mixed respectively, stationary for 5 min, then mixed, left at room temperature for 15 min, added to 24-well plate, shaken and cultured in incubator for 6 h, then replaced the medium, and cells were collected for double luciferase reporter gene detection after culture for 24 h.

After transfection for 24 h, the cell culture medium was removed, the cell lysate was added to 100 μL/ well, the cell lysate was dissolved at room temperature for 15 min, and the supernatant 80 μL was centrifuged at 12,000 rpm for 5 min, and the luciferase detection reagent 50 μL and the luciferase detection reagent 50 μL were added to the 24-well plate, respectively. The carrier luciferase activity and the sea cucumber luciferase activity were detected by ELIASA (MD-M5), and the ratio of the two was the relative luciferase activity.

### Xenograft model

Animal protocols have been approved by Institutional Animal Care and Use Committee (IACUC) and the Ethics Review Board of the Affiliated Kunshan Hospital of Jiangsu University (KSPH-ZAcuc-2024022). Severe combined immunodeficiency (SCID) female nude mice aged five-six weeks were purchased from the Animal Center of Jiangsu University and were raised indoors at standard conditions. Genetically modified CFPAC-1 cells were subcutaneously injected into the flanks of the nude mice. Each group consists of six mice, and the tumor volumes and mice weights were recorded every 5 days. In our study, mice were randomly allocated to experimental groups. Specifically, after tumor inoculation, mice were randomly assigned to the experimental groups using computer-generated random numbers (Excel RAND function). Mice were housed under identical conditions, and cages were balanced across groups to minimize potential bias. Mice were excluded if tumor establishment failed, if severe infection occurred, or if unexpected death unrelated to treatment was observed. Investigators were blinded to group allocation throughout the experiments and during outcome assessment. The immunohistochemistry (IHC) staining was conducted by the procedure described previously [33].

### Syngeneic model

Female C57BL/6 mice (6–8 weeks old) were obtained from Mairuisi Biotechnology Co., Ltd. (Nanjing, China) and maintained under specific pathogen-free (SPF) conditions with free access to food and water. All animal experiments were approved by Institutional Animal Care and Use Committee (IACUC) and the Ethics Review Board of the Affiliated Kunshan Hospital of Jiangsu University (KSPH-ZAcuc-2025009). Genetically modified PANC02 cells were subcutaneously injected into the right flank of each mouse to establish a syngeneic subcutaneous tumor model. Each group consists of six mice, and the tumor volumes and mice weights were recorded every 5 days. The randomization method and inclusion/exclusion criteria were the same as that used during the Xenograft model establishment. Multiplex Immunohistochemistry (mIHC) was conducted to show the tumor microenvironment (TME).

### Statistical analysis

Sample size for the in vivo experiments was determined based on pilot data. A priori power analysis was performed using the effect size estimated from preliminary tumor growth measurements (effect size d ≈ 1.3), with 80% power and a two-sided α of 0.05. The calculation indicated that at least 5 mice per group were required to detect the pre-specified effect size. Therefore, 6 mice per group were used to ensure adequate statistical power and to allow for potential experimental variability. In vitro experiments were repeated at least three times. Data were always with normal distribution and were presented as mean ± standard deviation (SD). Statistical analysis was performed using GraphPad Prism version 8.0.2 software and R. software (version 4.2.1). “limma” package, “clusterprofiler” package, “ggplot2” package, “survival” package, and “survminer” package were installed and used. Python software (version 3.12.4) was employed for scRNA-seq data analysis. Normality was evaluated using the Shapiro–Wilk test, and homogeneity of variance was assessed using Levene’s test. Comparative analysis between two distinct groups employed the two-tailed Student’s t-test. For comparisons involving more than two groups, One-way ANOVA with the Scheffe’ and Tukey Test was applied. All datasets subjected to t-tests or ANOVA met the assumption of equal variances. The difference was considered statistically significant as P value < 0.05.

## Results

### Bioinformatic analysis reveals elevated PRRG1 expression in PC

**Fig. 1:**
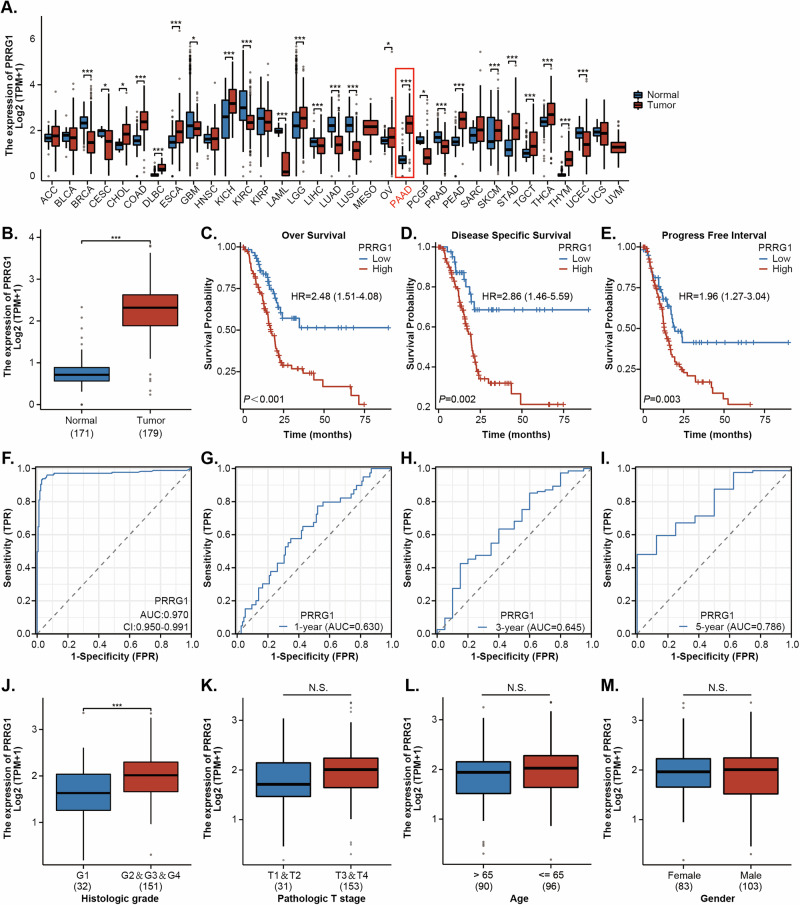
The bioinformatic analysis reveals high PRRG1 expression in PC. The PRRG1 expression in Pan-Cancer was shown.(**A**). The mRNA transcripts of PRRG1 in pancreatic cancer tissues (n = 179) compared with normal tissues (n = 171) in TCGA-GTEx database were shown (**B**). The Kaplan Meier Survival curve of overall survival (**C**), disease-specific survival (**D**), and progression-free interval (**E**) were performed based on PRRG1 expression in TCGA dataset. The ROC curves revealed that PRRG1 is a potential predictive marker for pancreatic cancer diagnosis (**F–I**). Subgroup analysis showed the relationship between PRRG1 expression and different clinical characteristics of the pancreatic cancer patients (**J–M**). “TPM” stands for transcripts per million, “AUC” is the area under the curve, “HR” represents the hazard rate. * *P* < 0.05, ** *P* < 0.01, *** *P* < 0.001, **** *P* < 0.0001. “N.S.” stands for non-statistical difference (*P* > 0.05).

Our comprehensive bioinformatic analysis demonstrated significant upregulation of PRRG1 in PC. Initial examination of pan-cancer data from the uniformly processed XENA database (UCSC Xena (xenabrowser.net)) revealed markedly increased PRRG1 expression in PC compared to other cancer types (Fig. 1A). To validate these findings, we analyzed PRRG1 transcript levels using data from The Cancer Genome Atlas (TCGA) database and Genotype-Tissue Expression (GTEx) projects. The analysis included 179 pancreatic tumor samples (“Tumor”) and 171 normal pancreatic tissue samples (“Normal”), which clearly showed that PRRG1 mRNA expression was significantly higher in PC tissues than in normal pancreatic tissues (Fig. 1B).

To further evaluate the clinical relevance of PRRG1 overexpression, we performed comprehensive prognostic analyses using TCGA data. Kaplan-Meier survival curves demonstrated that elevated PRRG1 expression was significantly associated with worse poor overall survival (hazard ratio [HR]: 2.48, *P* < 0.001) (Fig. 1C), disease-specific survival (HR: 2.86, *P* = 0.002) (Fig. 1D), and progress-free interval (HR: 1.96, *P* = 0.003) (Fig. 1E). Receiver operating characteristic (ROC) curve analysis indicated PRRG1’s strong diagnostic potential, with an area under the curve (AUC) of 0.970 (Fig. 1F). The AUC predicting prognosis were 0.630 for 1-year survival (Fig. 1G), 0.645 for 3-year survival (Fig. 1H) and 0.786 for 5-year survival (Fig. 1I), respectively. Subgroup analysis revealed that high PRRG1 expression correlated significantly with advanced histological grade (Fig. 1J), but showed no association with pathologic T stage (Fig. 1K), age (Fig. 1L) or gender (Fig. 1M). These findings collectively demonstrate that PRRG1 overexpression serves as both a diagnostic marker and an independent prognostic factor in PC, particularly associated with aggressive tumor biology and poor clinical outcomes.

### PRRG1 is upregulated in PC tissues and cell lines

**Fig. 2:**
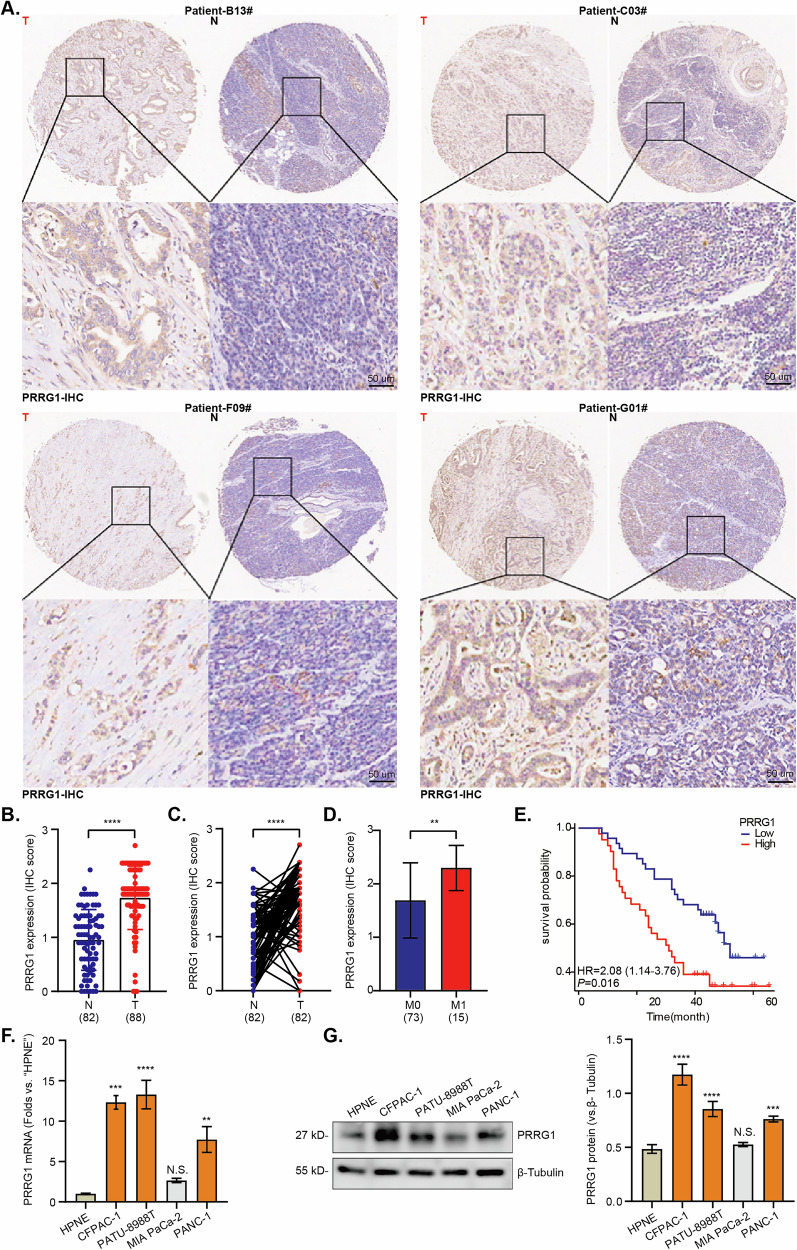
PRRG1 is upregulated in human PC tissues and different PC cells. Four groups of representative PRRG1 IHC images from pancreatic cancer tissues and normal pancreas tissues of patients (Patient -B13#/-C03#/-F09#/-G01#) were displayed in a tissue microarray (**A**). An unpaired PRRG1 IHC scores comparison (82“N” vs. 88“T”) (**B**) and a paired PRRG1 IHC scores comparison (82“N” vs. 82“T”) (**C**) were shown. PRRG1expression in pancreatic cancer tissues of patients with different metastasis status were compared (**D**). Kaplan-Meier survival analysis showed overall survival of patients with different PRRG1 levels (**E**). PRRG1 mRNA (**F**) and protein (**G**) expression in the established pancreatic cancer cells and normal human pancreatic ductal epithelial cells (HPNE) was tested by qRT-PCR and Western Blotting assays. The data were presented as mean ± standard deviation (SD). * *P* < 0.05 vs. “N” / “HPNE”. * *P* < 0.05, ** *P* < 0.01, *** *P* < 0.001, **** *P* < 0.0001. “N.S.” stands for non-statistical difference (*P* > 0.05). The representative PRRG1 IHC images of human tissue were shown. Scale bar = 50μm (**A**).

To validate PRRG1 expression patterns, we performed immunohistochemical (IHC) analysis using a tissue microarray containing 88 PC (“T”) and 82 adjacent normal pancreatic tissue (“N”) specimens. Representative IHC images from four representative cases (Patient -B13#/-C03#/-F09#/-G01#) clearly demonstrated higher PRRG1 expression in malignant compared to normal pancreatic tissues (Fig. 2A). Next, an evaluation of PRRG1 expression using IHC scores was conducted. Both unpaired (82“N” vs. 88“T”) and paired (82“N” vs. 82“T”) IHC scores comparison revealed significant elevation of PRRG1 expression in tumor versus normal tissues (Fig. 2B, C). Moreover, we found a higher PRRG1 levels in metastatic (M1) versus non-metastatic (M0) cases (Fig. 2D). Kaplan-Meier survival analysis demonstrated that increased PRRG1 expression was significantly associated with worse overall survival (Fig. 2E), further supporting its potential role as a prognostic biomarker in PC.

We analyzed both mRNA and protein levels across four established pancreatic cancer cell lines (CFPAC-1, PATU-8988T, MIA PaCa-2, and PANC-1) compared to normal human pancreatic ductal epithelial cells (HPNE) to systematically evaluate PRRG1 expression patterns. Compared with HPNE cell lines, qRT-PCR (Fig. 2F) and Western Blotting (Fig. 2G) confirmed a significantly higher mRNA and protein level of PRRG1 in CFPAC-1, PATU-8988T and PANC-1 cell lines. These consistent findings across multiple analytical platforms and cell lines robustly demonstrate that PRRG1 is frequently overexpressed in pancreatic cancer at both transcriptional and translational levels, corroborating our tissue-based observations.

### PRRG1 knockdown inhibits PC cell progression in vitro

**Fig. 3:**
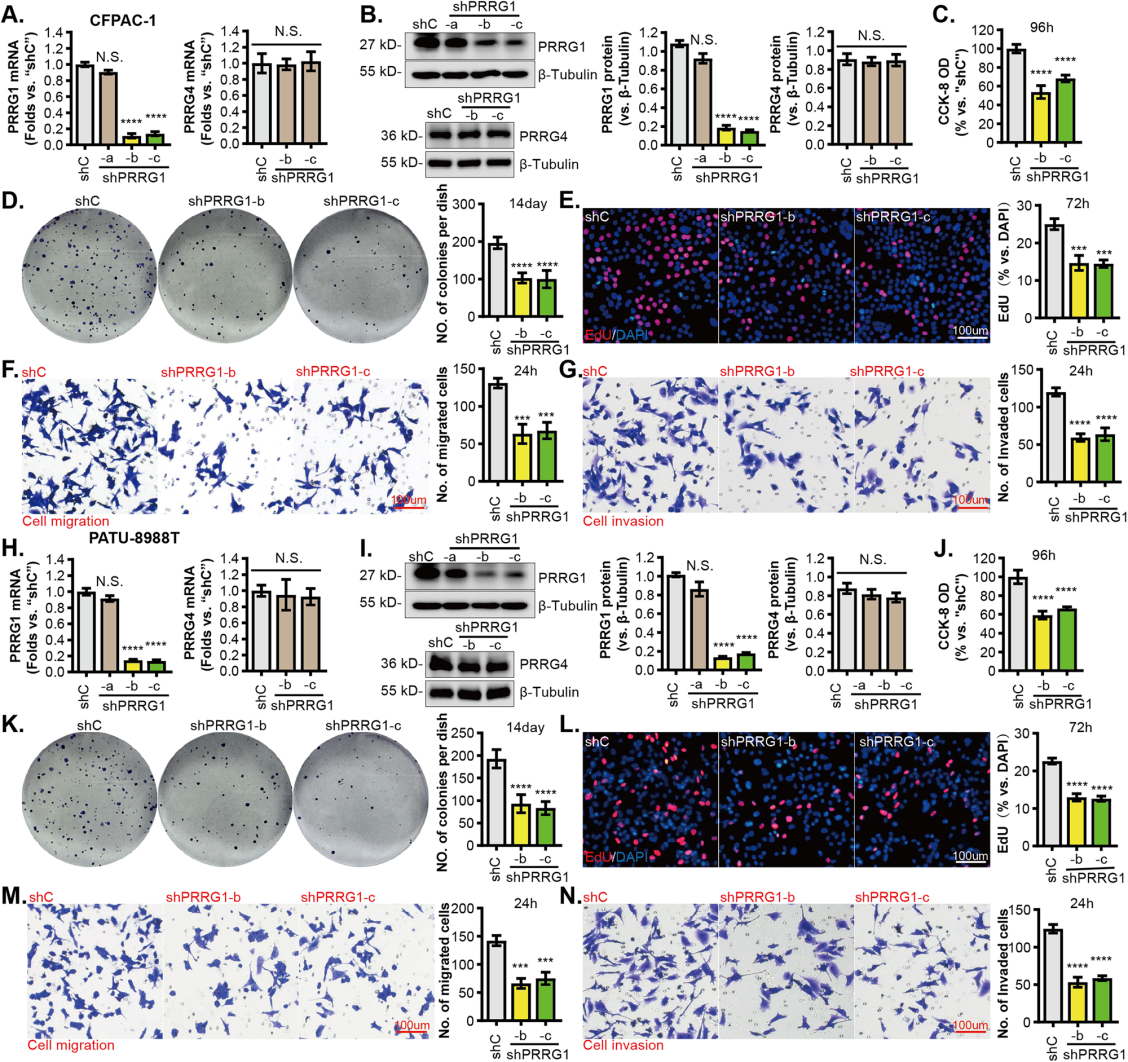
PRRG1 knockdown inhibits PC cell progression in vitro. Stable CFPAC-1 cells and PATU-8988T cells with PRRG1 shRNA (“shPRRG1-a/-b/-c”) or scramble nonspecific control shRNA (“shC”) were established and cultured. PRRG1 and PRRG4 expression in established cells were measured via qRT-PCR (**A** and **H**) and Western Blotting (**B** and **I**). CCK-8 assay was employed to test the cell viability. The OD value was detected at 96 h (**C** and **J**). Cell growth was tested by colony formation assay (**D** and **K**) and EdU assay (**E** and **L**). Cell migration and invasion were tested by Transwell (**F** and **M**) and Matrigel Transwell assays (**G** and **N**). Experiments in this figure were repeated at least three times (n = 3, biological repeats). The data were presented as mean ± standard deviation (SD). * *P* < 0.05 vs. “shC”. * *P* < 0.05, ** *P* < 0.01, *** *P* < 0.001, **** *P* < 0.0001. “N.S.” stands for non-statistical difference (*P* > 0.05). Scale bar = 100μm.

Based on our finding of PRRG1 overexpression in PC cells, we investigated its functional role through lentivirus-mediated gene silencing in CFPAC-1 cells. Three distinct shRNA constructs targeting non-overlapping PRRG1 sequences (“shPRRG1-a/-b/-c”) and a scrambled control (“shC”) were used to establish stable knockdown cell lines following puromycin selection. In stable shPRRG1-b/shPRRG1-c-expressing CFPAC-1 cells, PRRG1 mRNA and protein levels were both reduced significantly than in control cells, whereas PRRG4 mRNA and protein levels remained unchanged (Fig. 3A and B). CCK-8 assay results revealed significant reduction in PRRG1-silenced CFPAC-1 cell viability at 96 h post-transfection (Fig. 3C). Meanwhile, PRRG1 knockdown significantly inhibited CFPAC-1 cell proliferation, proved by the decreased results of cell colony formation assay (Fig. 3D) and EdU /DAPI double staining assay (Fig. 3E). The “Transwell” (Fig. 3F) and “Matrigel Transwell” (Fig. 3G) assays revealed that PRRG1 knockdown potently inhibited migration and invasion ability of CFPAC-1 cell in vitro, respectively.

PATU-8988T cells were also processed with shRNA-induced PRRG1 silencing. Both mRNA and protein levels of PRRG1 instead of PRRG4 were found decreased significantly in shPRRG1-b/shPRRG1-c-expressing PATU-8988T cells than in control cells (Fig. 3H, I). The results demonstrated consistent anti-tumor effects across multiple functional assays. Decreased cell viability was confirmed in PRRG1 knockdown PATU-8988T cells by CCK-8 assay (Fig. 3J). PRRG1 silencing significantly inhibited PATU-8988T cell proliferation by reducing colony formation (Fig. 3K) and EdU-positive nuclei ratio (Fig. 3L). Moreover, PRRG1 knockdown in PATU-8988T cells significantly inhibited cell migration (Fig. 3M) and invasion (Fig. 3N). These consistent results across two distinct PC cell lines (CFPAC-1 and PATU-8988T) provide compelling evidence that PRRG1 plays a critical role in maintaining the malignant phenotype of PC cells, affecting multiple oncogenic processes including proliferation, migration and invasion.

### Ectopic overexpression of PRRG1 induces pro-cancerous activity in vitro

**Fig. 4:**
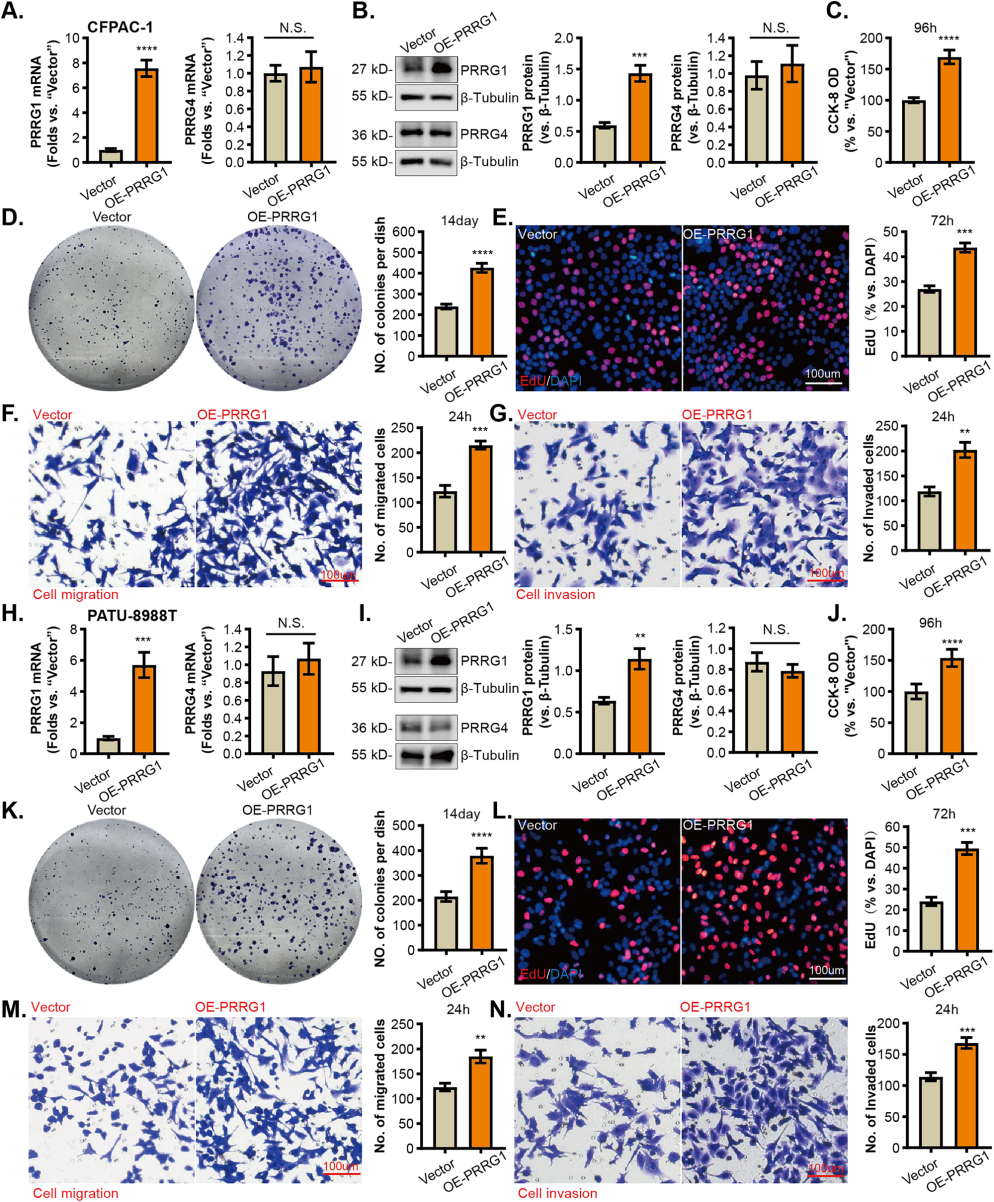
Ectopic overexpression of PRRG1 induces pro-cancerous activity in PC cells. Stable CFPAC-1 cells and PATU-8988T cells with PRRG1 cDNA-expressing lentiviral construct (“OE-PRRG1”) or empty vector (“Vector”) were established and cultured. PRRG1 and PRRG4 expression in the established cells were measured by qRT-PCR (**A** and **H**) and Western Blotting (**B** and **I**). Cell viability (**C** and **J**), colony formation (**D** and **K**), proliferation (**E** and **L**), in vitro cell migration (**F** and **M**) and invasion (**G** and **N**) were tested by assays as described. Experiments in this figure were repeated at least three times (n = 3, biological repeats). The data were presented as mean ± standard deviation (SD). * *P* < 0.05 vs. “shC”. * *P* < 0.05, ** *P* < 0.01, *** *P* < 0.001, **** *P* < 0.0001. “N.S.” stands for non-statistical difference (*P* > 0.05). Scale bar = 100μm.

To complement our loss-of-function studies, we investigated the oncogenic potential of PRRG1 through gain-of-function experiments in PC cells. CFPAC-1 cells were transfected with the lentiviral particles encoding PRRG1 cDNA (“OE-PRRG1”). Control cells were transfected with the empty vector (“Vector”). Stable polyclonal populations were selected using puromycin. The PRRG1 mRNA and protein levels were both significantly elevated in stable OE-PRRG1-expressing CFPAC-1 cells, while PRRG4 mRNA and protein levels remained unchanged (Fig. 4A, B). Measured by the CCK-8 viability assay, PRRG1 overexpression increased CFPAC-1 cell viability compared with control cells (Fig. 4C). Colony formation capacity (Fig. 4D) and EdU incorporation (Fig. 4E) were significantly increased in PRRG1-overexpressed CFPAC-1 cell. Moreover, the “Transwell” (Fig. 4F) and “Matrigel Transwell” (Fig. 4G) assays showed increased migratory and invasive ability in PRRG1-overexpressed CFPAC-1 cell in vitro, respectively.

Treatment with the same lentiviral particles in PATU-8988T cells led to significantly upregulation of PRRG1 mRNA and protein levels, without affecting PRRG4 mRNA and protein levels (Fig. 4H, I). Consistent with the results observed in CFPAC-1 cells, lentiviral-mediated PRRG1 overexpression in PATU-8988T cells demonstrated similar pro-tumorigenic effects. Increased CCK-8 viability OD (Fig. 4J), colony formation (Fig. 4K) and EdU incorporation (Fig. 4L) revealed enhanced proliferative capacity in PRRG1-overexpressed PATU-8988T cell. In addition, increased metastatic potential, induced by ectopic PRRG1 overexpression, was confirmed by increased cell migration (Fig. 4M) and invasion (Fig. 4N). These findings in PATU-8988T cells, together with the results from CFPAC-1 cells, provide compelling evidence that PRRG1 overexpression is sufficient to drive multiple aspects of PC progression, including enhanced proliferation, migration and invasion in vitro.

### PRRG1 promotes PC cell progression by activating PI3K-Akt cascade

**Fig. 5:**
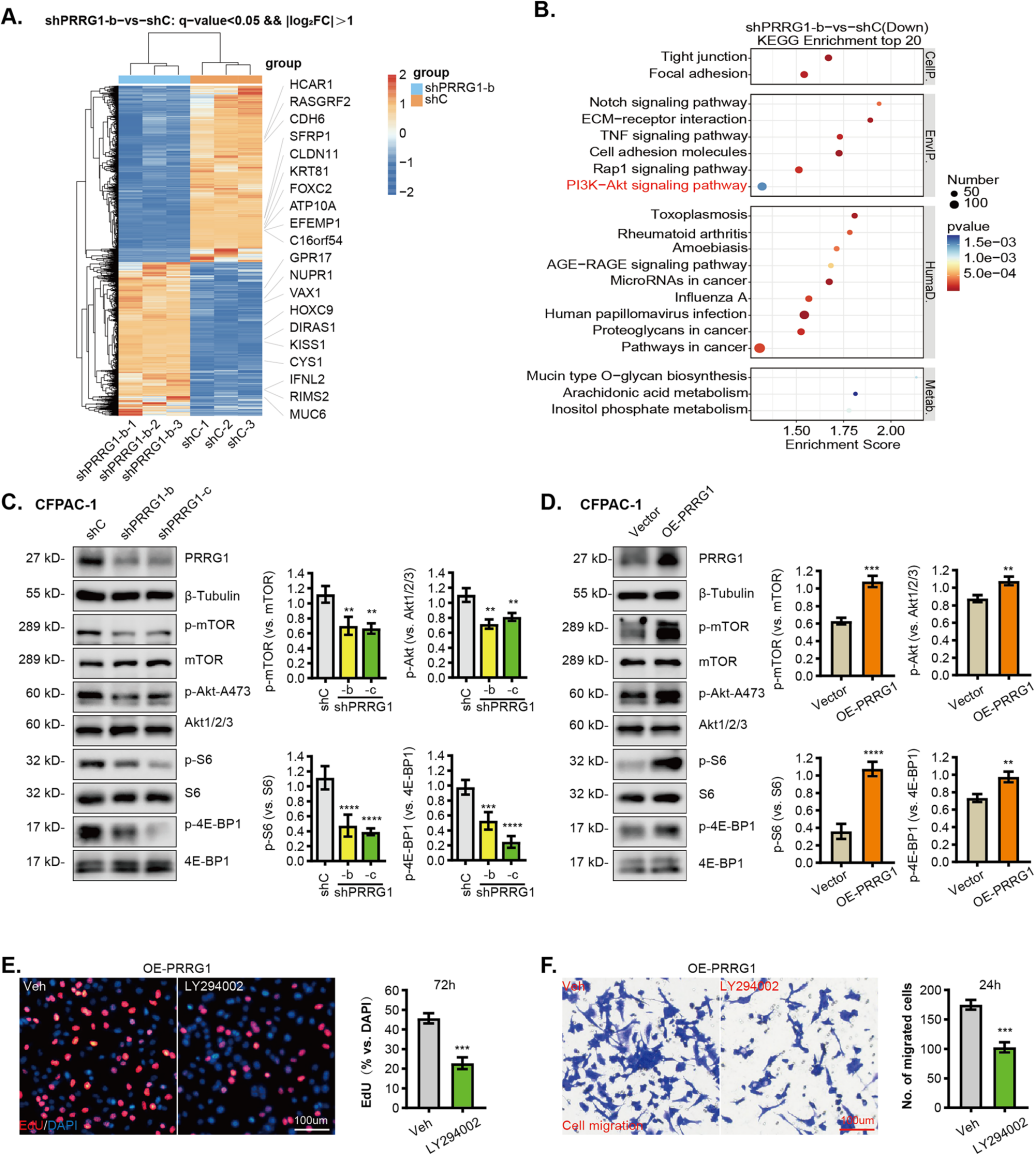
PRRG1 promotes PC cell progression by activating PI3K-Akt cascade. Differentially expressed genes (DEGs) between PRRG1-silenced CFPAC-1 cells (“shPRRG1-b”) and control CFPAC-1 cells (“shC”) were analyzed by RNA-sequencing (RNA-seq), shown as the heatmap (**A**). KEGG enrichment analysis of PRRG1-associated DEGs was shown in the bubble plot (**B**). PRRG1 knockdown inhibited phosphorylation of mTOR, Akt (Ser-473), S6 and 4E-BP1 in CFPAC-1 cells (**C**). Ectopic overexpression of PRRG1 significantly increased the phosphorylation of mTOR, Akt (Ser-473), S6 and 4E-BP1 in CFPAC-1 cells (**D**). In “OE-PRRG1” CFPAC-1 cells treated with the PI3K-Akt inhibitor LY294002 (5um) or the vehicle control (0.1% DMSO, “Veh”) for indicated time periods, EdU-positive nuclei ratio (**E**) and cell migration ability (**F**) were tested. Experiments in this figure were repeated at least three times (n = 3, biological repeats). The data were presented as mean ± standard deviation (SD). *P < 0.05 versus “shC” / “Vector” / “Veh”. * *P* < 0.05, ** *P* < 0.01, *** *P* < 0.001, **** *P* < 0.0001. Scale bar = 100 μm.

The molecular mechanisms underlying PRRG1-mediated oncogenesis are still unknown. Therefore, RNA-sequencing (RNA-seq) was performed to analyze PRRG1-associated differentially expressed genes (DEGs) between PRRG1-silenced (“shPRRG1-b”) and control (“shC”) CFPAC-1 cells (Fig. 5A). KEGG pathway enrichment analysis demonstrated PRRG1-associated DEGs were enriched in PI3K-Akt signaling pathway (Fig. 5B). To identify if PRRG1 involved in activating PI3K-Akt signaling pathway, we tested the phosphorylation levels of several markers in this pathway. The Western Blotting results confirmed that PRRG1 knockdown inhibited phosphorylation of mTOR, Akt (Ser-473), S6 and 4E-BP1 in CFPAC-1 cells (Fig. 5C). Contrarily, PRRG1 overexpression increased phosphorylation of these markers (Fig. 5D). Moreover, in “OE-PRRG1” CFPAC-1 cells, treatment with LY294002, a PI3K-Akt pathway inhibitor [34], largely decreased EdU incorporation (Fig. 5E) and cell migration in vitro (Fig. 5F).Together, these results strongly support PI3K-Akt signaling as the predominant mechanistic pathway through which PRRG1 exerts its oncogenic effects in PC cells.

### KLF4 serves as a critical transcription factor of PRRG1 in PC

**Fig. 6:**
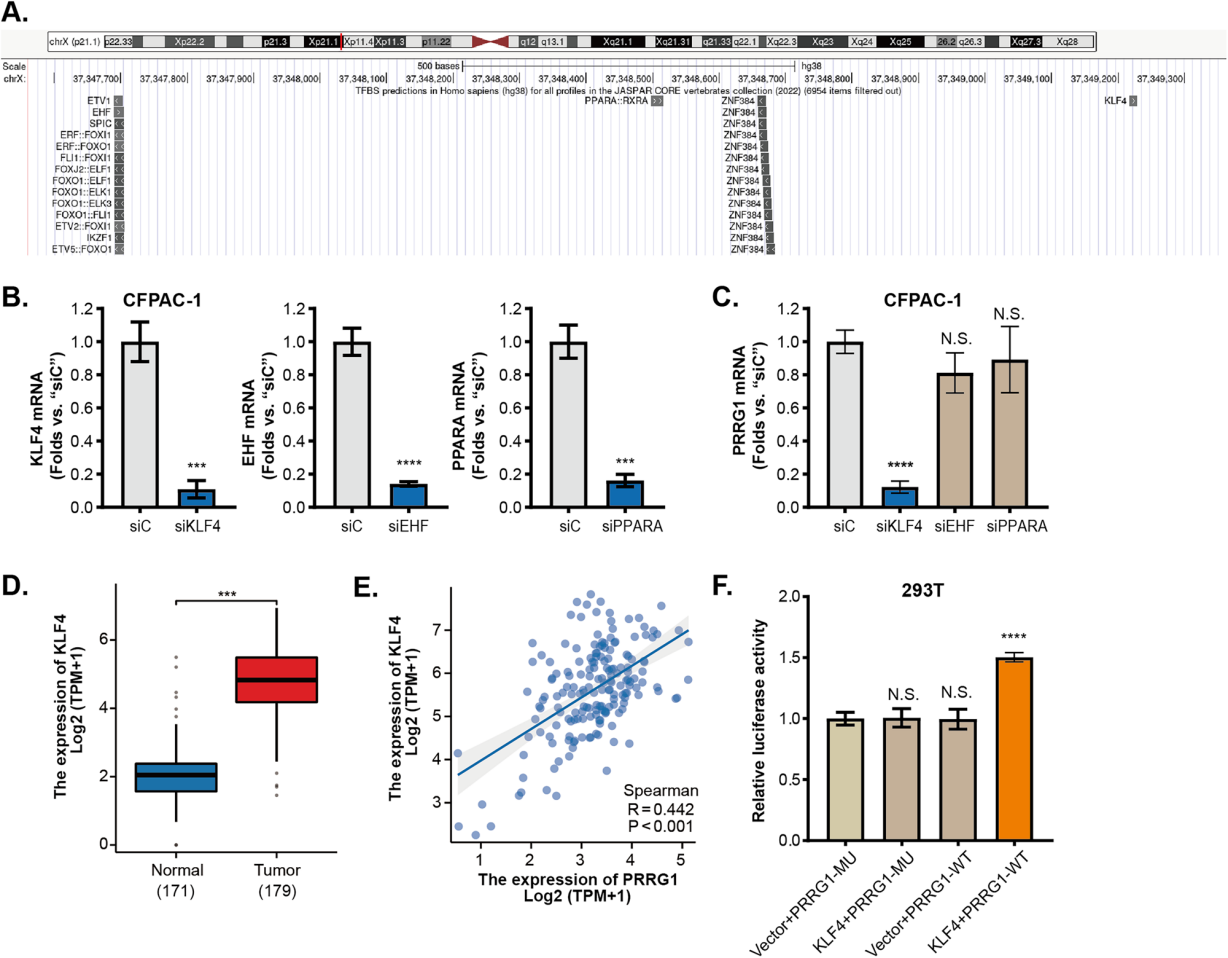
KLF4 is a key transcription factor of PRRG1 in PC cells. The JASPAR transcription factor database was employed to explore transcription factors with highest predicted binding affinity to PRRG1 promoter (**A**). After CFPAC-1 cells transfected with specific siRNAs targeting these transcription factors, decreased mRNA levels of them were shown (**B**). PRRG1 mRNA levels were reduced in CFPAC-1 cells transfected with siKLF4 (**C**). The mRNA transcripts of PRRG1 in pancreatic cancer tissues (n = 179) compared with normal tissues (*n* = 171) in TCGA-GTEx database were shown (**D**). The Scatter plot of correlation between the expression of PRRG1 and KLF4 was displayed (**E**). The Dual-Luciferase Reporter Assay results showed that KLF4 was significantly bind with PRRG1 promoter (**F**). Experiments in this figure were repeated at least three times (*n* = 3, biological repeats). “TPM” stands for transcripts per million, *P < 0.05 versus “siC” / “Vector+PRRG1-MU”. * *P* < 0.05, ** *P* < 0.01, *** *P* < 0.001, **** *P* < 0.0001. “N.S.” stands for non-statistical difference (*P* > 0.05).

Since PRRG1 is overexpressed in PC cells, we investigated its transcriptional regulation. JASPAR database analysis identified three transcription factors with high predicted binding affinity to the PRRG1 promoter: KLF4, EHF and PPARA (Fig. 6A). CFPAC-1 cell lines were successfully transfected with specific siRNAs targeting these transcription factors and siRNA-mediated knockdown achieved, confirmed by the decreased mRNA levels of these transcription factors (Fig. 6B). While, we only found siKLF4 significantly reduced PRRG1 mRNA levels in CFPAC-1 cells (Fig. 6C). Bioinformatic analysis based on TCGA database revealed that KLF4 is upregulated in PC (Fig. 6D). The Scatter plot further supported that relationship between the expression of PRRG1 and KLF4 are significant correlated (R = 0.442, *P* < 0.001) (Fig. 6E). Moreover, the Dual-Luciferase Reporter Assay results demonstrated direct KLF4 binding to the PRRG1 promoter (Fig. 6F). Above results revealed that KLF4 regulate PRRG1 expression at the mRNA level, as a key transcription factor bind with PRRG1 promoter. The KLF4-PRRG1 regulatory axis could be a novel mechanistic pathway contributing to PC progression.

### PRRG1 knockdown suppresses PC cell growth in vivo

**Fig. 7:**
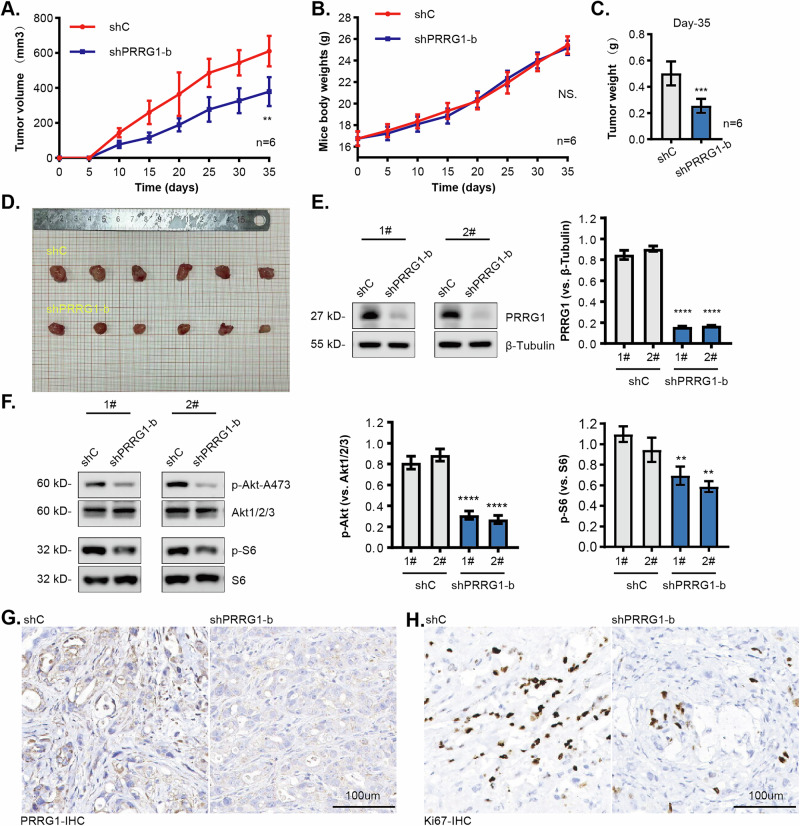
PRRG1 knockdown inhibits PC cell growth in vivo*.* Six mice a group, per nude mice was subcutaneously injected with six million PRRG1-silenced CFPAC-1 cells (“shPRRG1-b”) or control CFPAC-1 cells (“shC”) to establish nude mice xenograft model. The shPRRG1-b xenografts grew significantly slower than the shC xenografts (**A**). Mice weights of two groups were found no significant difference (**B**). The shPRRG1-b xenografts were significantly lighter than shC xenografts (**C**). Size of two groups of xenografts was measured (**D**). Two pairs of xenografts were chosen (-1#/-2#), compared with shC xenografts, PRRG1 protein (**E**) levels as well as phosphorylation of Akt and S6 (**F**) were significantly reduced in shPRRG1-b xenografts. The IHC results confirmed significantly downregulated of PRRG1 (**G**) and Ki67 (**H**) expression in shPRRG1-b xenografts. The data were presented as mean ± standard deviation (SD, *n* = 6). *P < 0.05 versus “shC”. * *P* < 0.05, ** *P* < 0.01, *** *P* < 0.001, **** *P* < 0.0001. “N.S.” stands for non-statistical difference (*P* > 0.05). Scale bar = 100 μm.

To investigate the role of PRRG1 in PC progression in vivo, we established a nude mice xenograft model. Each mouse was subcutaneously injected with six million CFPAC-1 cells stably expressing either PRRG1 shRNA (“shPRRG1-b”) or a control shRNA (“shC”). The study included six mice per group, and tumor volumes and body weights were monitored every five days. Notably, the shPRRG1-b xenografts exhibited significantly slower growth compared to the shC xenografts (Fig. 7A), while no significant difference in body weight was observed between the two groups (Fig. 7B). At the endpoint (Day 35), all mice were euthanized, and the xenografts were excised and weighed. The shPRRG1-b tumors were significantly lighter than the control tumors (Fig. 7C), and their sizes were significantly smaller (Fig. 7D). To validate PRRG1 knockdown at the molecular level, we analyzed two representative xenograft pairs (-1#/-2#). Western blotting confirmed a significant reduction in PRRG1 protein expression in shPRRG1-b tumor tissues compared to controls (Fig. 7E). Additionally, phosphorylation levels of Akt and S6 were markedly decreased in the shPRRG1-b group (Fig. 7F). Immunohistochemical (IHC) staining further supported these findings, demonstrating downregulation of both PRRG1 (Fig. 7G) and the proliferation marker Ki67 (Fig. 7H) in shPRRG1-b xenografts. Collectively, these results demonstrate that PRRG1 knockdown effectively suppresses PC cell growth in vivo, highlighting its role in promoting tumor progression.

### PRRG1 overexpression enhances PC cell growth in vivo

**Fig. 8:**
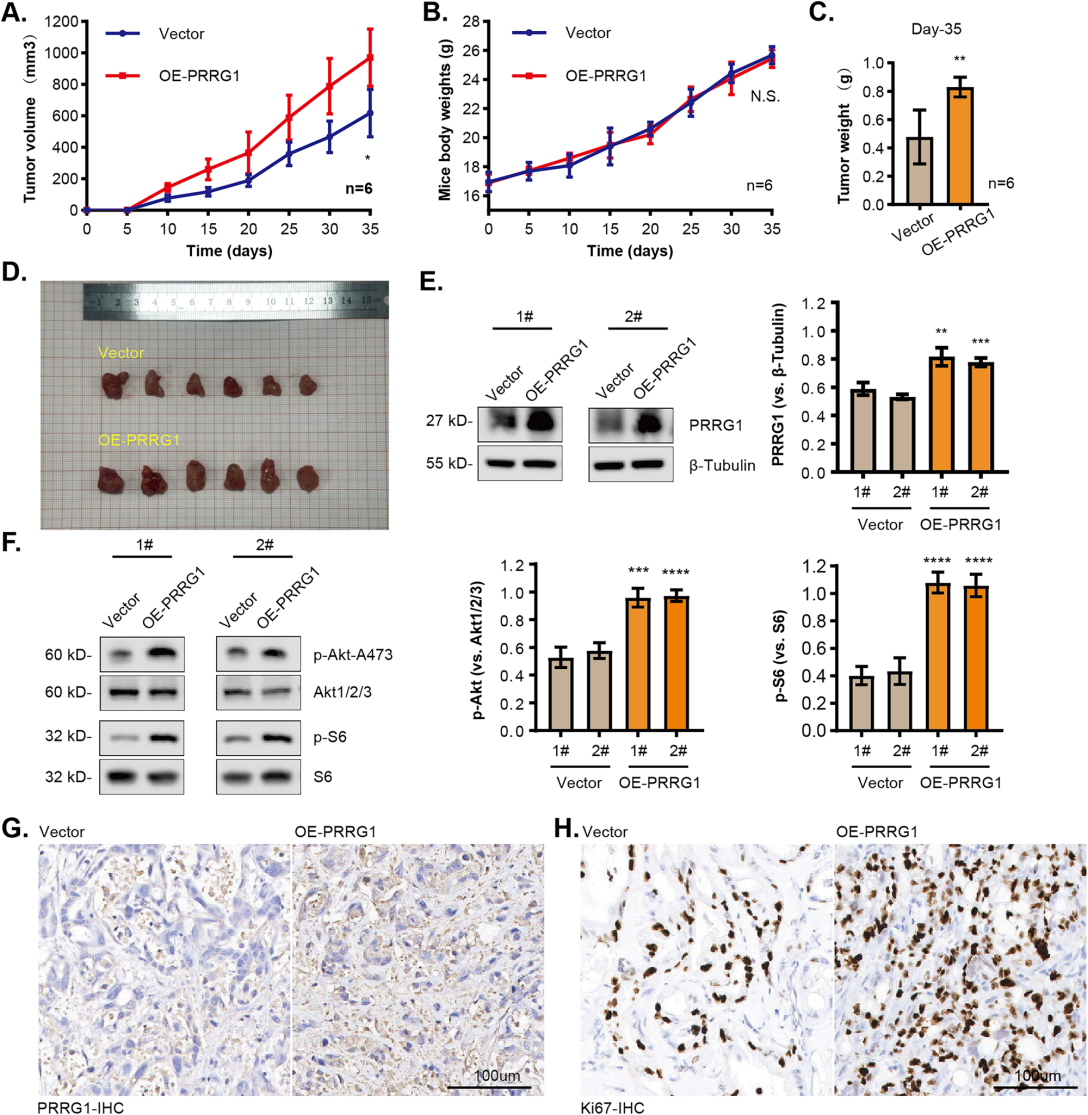
PRRG1 overexpression promotes PC cell growth in vivo*.* To establish nude mice xenograft model, per nude mice was subcutaneously injected with six million PRRG1-overexpressed CFPAC-1 cells (“OE-PRRG1”) or control CFPAC-1 cells (“Vector”) in six mice a group. The OE-PRRG1 xenografts grew significantly faster than the Vector xenografts (**A**). Mice weights of two groups were found no significant difference (**B**). The OE-PRRG1 xenografts were significantly heavier than Vector xenografts (**C**). Size of two groups of xenografts was measured (**D**). Two pairs of xenografts were chosen (-1#/-2#), compared with Vector xenografts, PRRG1 protein (**E**) levels as well as phosphorylation of Akt and S6 (**F**) were significantly elevated in OE-PRRG1 xenografts. The IHC results confirmed significantly upregulated of PRRG1 (**G**) and Ki67 (**H**) expression in OE-PRRG1 xenografts. The data were presented as mean ± standard deviation (SD, *n* = 6). *P < 0.05 versus “Vector”. * *P* < 0.05, ** *P* < 0.01, *** *P* < 0.001, **** *P* < 0.0001. “N.S.” stands for non-statistical difference (*P* > 0.05). Scale bar = 100 μm.

In the next in vivo experiment, we established a xenograft model by subcutaneously injecting six million CFPAC-1 cells overexpressing PRRG1 (“OE-PRRG1”) or control vector-transfected cells (“Vector”) into nude mice (n = 6 per group). Tumor volumes and body weights were monitored every five days over a 35-day observation period. Notably, the OE-PRRG1 xenografts exhibited significantly accelerated growth compared to the Vector group (Fig. 8A), while no significant difference in body weight was observed between the two groups (Fig. 8B). At the endpoint, the tumors were excised and weighed, revealing that the OE-PRRG1 xenografts were significantly heavier than the Vector controls (Fig. 8C). Representative images further illustrated the size difference between the two groups (Fig. 8D). Western blot analysis of two selected xenograft pairs (-1#/-2#) confirmed elevated PRRG1 protein levels in the OE-PRRG1 group (Fig. 8E), along with increased phosphorylation of Akt and S6 (Fig. 8F), suggesting enhanced oncogenic signaling. Consistent with these findings, immunohistochemical (IHC) staining demonstrated significant upregulation of both PRRG1 (Fig. 8G) and the proliferation marker Ki67 (Fig. 8H) in OE-PRRG1 xenografts. Taken together, these results demonstrate that PRRG1 overexpression drives PC progression in vivo by promoting tumor growth and activating key oncogenic pathways.

### Warfarin suppresses the pro-tumorigenic effects driven by PRRG1 overexpression in vitro and in vivo

**Fig. 9:**
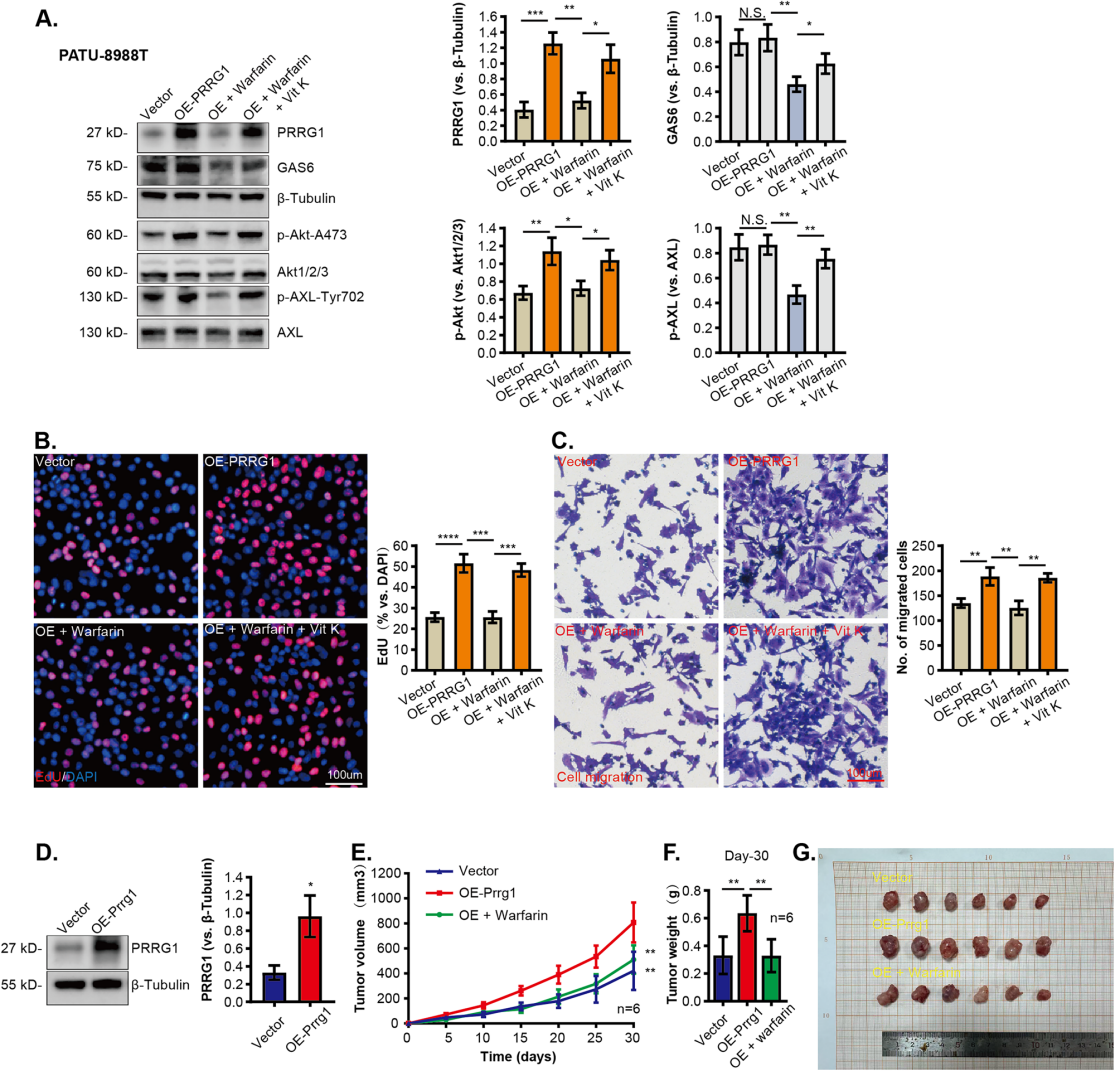
Warfarin suppresses the pro-tumorigenic effects driven by PRRG1 overexpression in vitro and in vivo*.* In “OE-PRRG1” PATU-8988T cells, the protein levels of PRRG1 and GAS6, as well as the phosphorylation levels of Akt and AXL, were reduced following treatment with a low dose of warfarin (2 µM), but they were restored after a vitamin K (25 µM) supplementation experiment (**A**). As demonstrated by Edu (**B**) and Transwell (**C**) assays, the proliferative and migratory abilities of “OE-PRRG1” cells were inhibited by a low dose of warfarin (2 µM) and could be restored by vitamin K (25 µM). Western blot analysis showed that PRRG1 protein levels were significantly increased in “OE- Prrg1” PANC02 cells compared with “Vector” cells (**D**). In vivo syngeneic model experiment suggested that the “OE-Prrg1” models grew significantly faster than the “Vector” models, whereas low-dose warfarin markedly suppressed tumor growth in the “OE + warfarin” models (**E**). “OE-Prrg1” models were significantly heavier than those in the “Vector” group, while models in the “OE + warfarin” group were significantly lighter than those in the “OE-Prrg1” group (**F**). Size of three groups of models was measured (**G**). In vitro Experiments in this figure were repeated at least three times (n = 3, biological repeats). The data were presented as mean ± standard deviation (SD). * *P* < 0.05 vs. “Vector”. * *P* < 0.05, ** *P* < 0.01, *** *P* < 0.001, **** *P* < 0.0001. “N.S.” stands for non-statistical difference (*P* > 0.05). Scale bar = 100μm.

Warfarin acts as an inhibitor of vitamin K–dependent (VKD) proteins through suppression of vitamin K hydroquinone production, leading to inhibition of γ-glutamyl carboxylation [19, 20]. Treatment with low-dose warfarin (2 μM, a dose insufficient to affect coagulation) inhibited VKD proteins from forming Gla residues and consequently decreased the levels of VKD proteins [11, 30]. In PRRG1-overexpressing PATU-8988T cells, low-dose warfarin (2 μM) decreased levels of PRRG1 and Gas6, accompanied by a reduction in phosphorylated Akt and AXL (Fig. 9A). Notably, these warfarin-induced inhibitory effects were reversed by the addition of vitamin K. The total protein levels of Akt and AXL showed little change in these experiments. Moreover, PRRG1 overexpression did not significantly elevate the expression levels of the GAS6/AXL axis. In vitro, treatment with low-dose warfarin suppressed the proliferation (Fig. 9B) and migration (Fig. 9C) of PRRG1-overexpressing PATU-8988T cells, whereas supplementation with vitamin K restored these abilities.

In vivo, a subcutaneous syngeneic model was established by injecting six million PANC02 cells overexpressing Prrg1 (“OE- Prrg1”) or control vector–transfected cells (“Vector”) into the axillary region of per female C57BL/6 mice to generate subcutaneous tumors. Western blot analysis showed that PRRG1 protein levels were significantly increased in “OE- Prrg1” PANC02 cells compared with “Vector” cells (Fig. 9D). Among the mice inoculated with “OE- Prrg1” cells (*n* = 12), half were randomly assigned to the warfarin treatment group (“OE + warfarin”, *n* = 6), which received drinking water containing a low dose of warfarin (2 μmol/L) starting 48 h after cell inoculation. The remaining mice (“OE- Prrg1”, *n* = 6) were received regular drinking water without warfarin. The “Vector” group (*n* = 6) was used as the normal control. Tumor volumes and body weights were monitored every five days over a 30-day observation period. Notably, the subcutaneous tumors in the “OE- Prrg1” group exhibited a significantly faster growth rate compared with those in the “Vector” group, while tumor growth was markedly suppressed in the “OE + warfarin” group (Fig. 9E). At the endpoint, the tumors were excised and weighed. The subcutaneous tumors in the “OE- Prrg1” group were significantly heavier than those in the “Vector” group; however, warfarin treatment markedly reduced the tumor weight in the “OE- Prrg1” group. (Fig. 9F). Representative images further illustrated the size difference between these three groups (Fig. 9G). Collectively, these results suggested that low-dose warfarin inhibits the pro-cancerous activity of PC cells induced by PRRG1 overexpression in vitro and in vivo.

### PRRG1 plays a pivotal role in PC tumor microenvironment

**Fig. 10:**
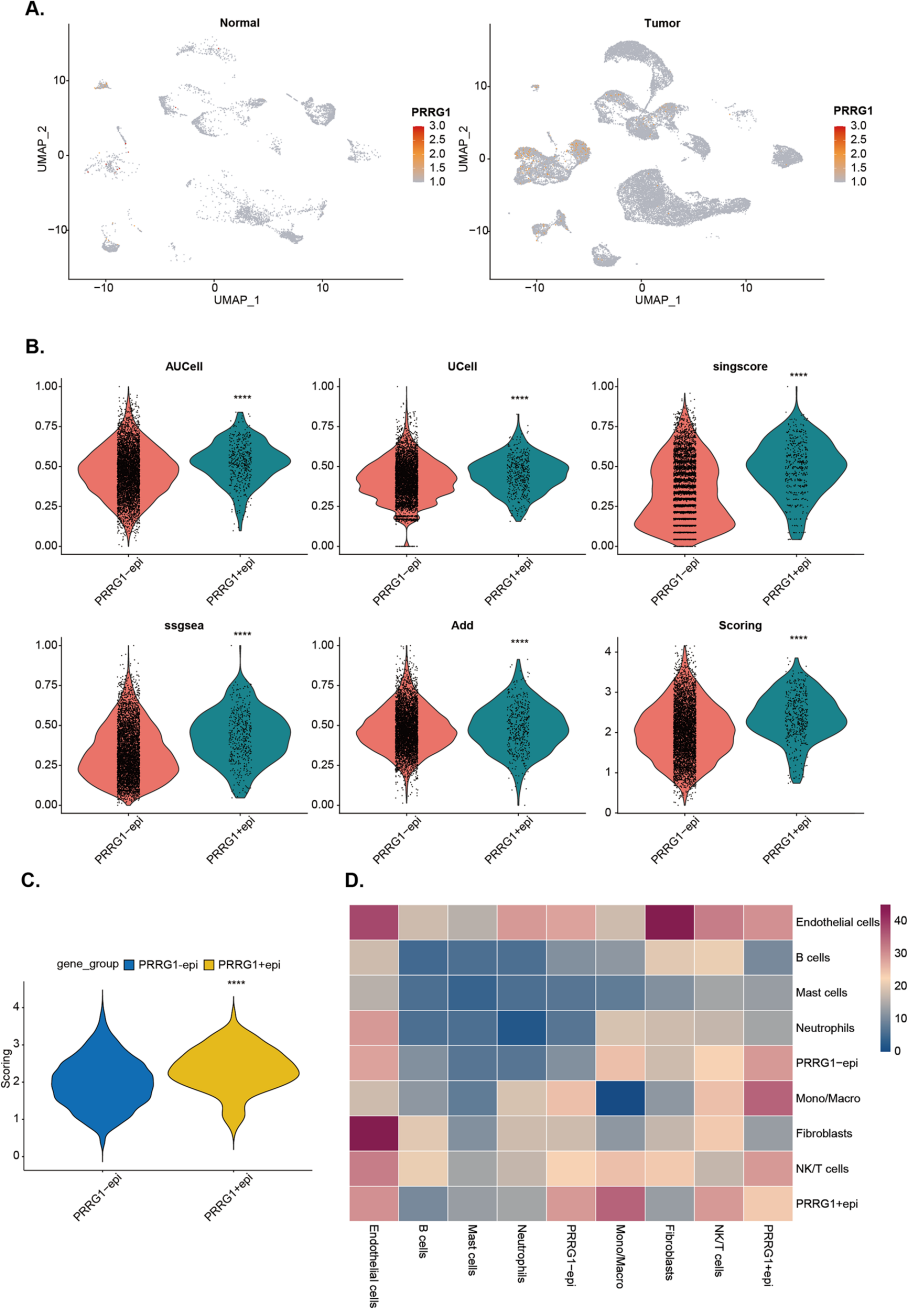
PRRG1 plays a key role in PC tumor microenvironment. PRRG1 expression of different cellular subsets in normal and tumor samples were demonstrated by UMAP chart (**A**). Score in regard to PI3K-Akt signaling pathway activity based on the calculation of different cell scoring algorithms were shown by violin plot (**B** and **C**). After divided PC epithelial cells into PRRG1-negative group and PRRG1-positive group, communication between different cellular subsets were demonstrated by heat map (**D**), *P < 0.05 versus “PRRG1 - epi”. * *P* < 0.05, ** *P* < 0.01, *** *P* < 0.001, **** *P* < 0.0001.

To investigate PRRG1’s role in the PC tumor microenvironment, we analyzed single-cell RNA sequencing (scRNA-seq) data from the GEO dataset GSE155698. Initial quality control revealed a strong correlation between UMI counts and mRNA levels, while mitochondrial gene expression showed no such correlation (Fig. S1A). After excluding low-quality cells (Fig. S1B, C), we identified the top 2000 highly variable genes (Fig. S1D) and performed principal component analysis (PCA, Fig. S1E). Since PCA failed to clearly segregate different cell types, we employed t-SNE and UMAP algorithms, which successfully classified the cells into 32 distinct clusters (Fig. S2A). Using CellMarker 2.0 [35] as reference, we annotated these clusters based on established biomarkers (Fig. S2B). The resulting cellular composition included: T cells (clusters 0, 1, 4, 21), macrophages (cluster 2), B cells (clusters 9, 15, 30), monocytes (clusters 7, 10, 12, 27, 29), NK cells (clusters 3, 5, 11, 13, 31), epithelial cells (clusters 6, 8, 14, 16, 17, 25, 28), endothelial cells (cluster 26), neutrophils (clusters 3, 18, 19), smooth muscle cells (cluster 20), and tissue stem cells (clusters 22, 23) (Fig. S2C, D). Notably, comparative analysis revealed significantly higher PRRG1 expression in tumor-derived epithelial cells compared to their normal counterparts (Fig. 10A, Fig. S2E). Further stratification of PC epithelial cells based on PRRG1 expression identified PRRG1-positive (453 cells) and PRRG1-negative (5097 cells) populations. Using five distinct scoring algorithms (AUCell, UCell, singscore, ssgsea, Add), we demonstrated enhanced PI3K-Akt signaling pathway activity in PRRG1-positive epithelial (“PRRG1 + epi”) cells (Fig. 10B, C).

**Fig. 11:**
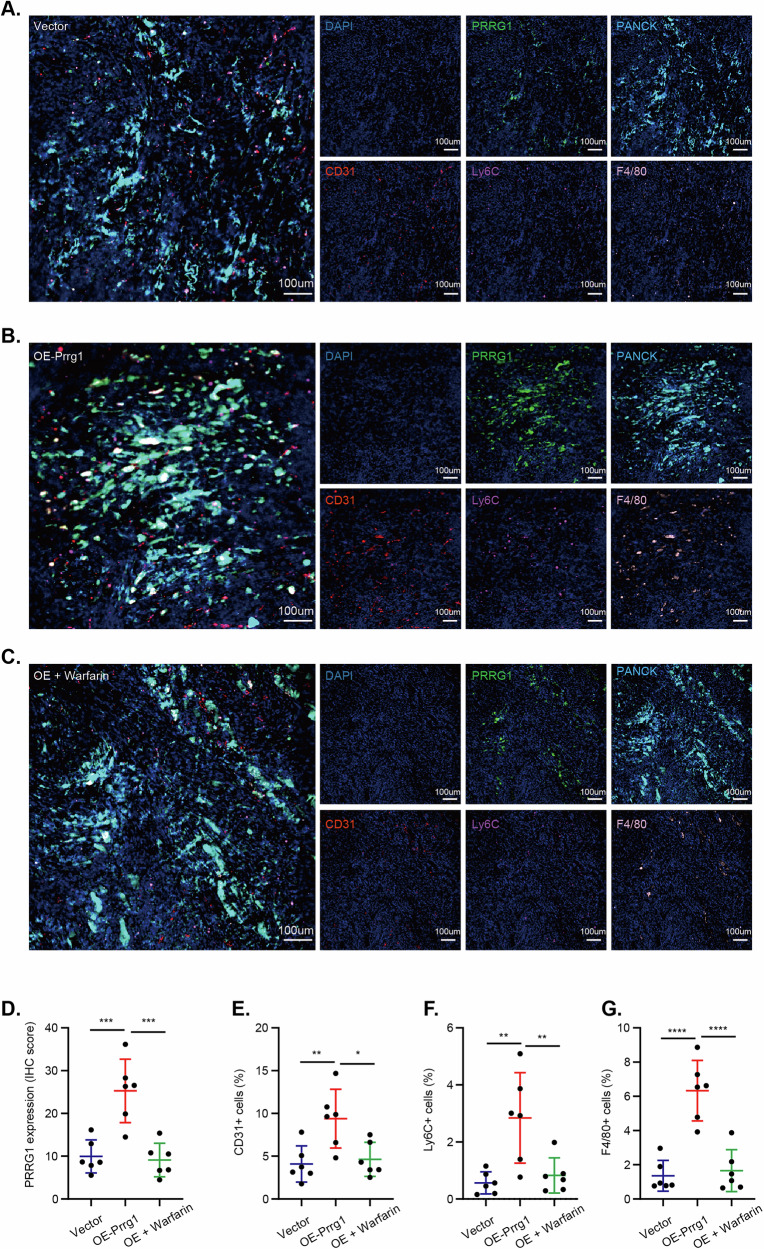
PRRG1 overexpression alters the immune microenvironment. Representative mIHC images of immunocompetent model sections from the “Vector” (**A**), “OE-Prrg1” (**B**) and “OE + warfarin” (**C**) groups are shown, with each color panel shown alongside. Various markers were visualized by distinct colors, including PRRG1 in green, PANCK in blue, CD31 in red, Ly6C in purple and F4/80 in pink, with DAPI used for counterstaining. PRRG1 expression (IHC score) (**D**), accompanied by a marked increase in the proportions of CD31⁺ (**E**), Ly6C⁺ (**F**) and F4/80⁺ (**G**) cells were significantly higher in the “OE-Prrg1” group than in the “Vector” group, while warfarin reversed the alterations induced by PRRG1 overexpression in TME. The data were presented as mean ± standard deviation (SD, n = 6). * *P* < 0.05 vs. “Vector”. * *P* < 0.05, ** *P* < 0.01, *** *P* < 0.001, **** *P* < 0.0001. Scale bar = 100μm.

Cell-cell communication analysis revealed that PRRG1-positive epithelial cells exhibited significantly stronger interactions with mono/macro cells and endothelial cells compared to PRRG1-negative epithelial (“PRRG1 - epi”) cells (Fig. 10D, Fig. S3A, B). Multiplex IHC analysis of immunocompetent C57BL/6 mice models revealed that, compared with the “Vector” group, tumors from the “OE-Prrg1” group exhibited markedly increased PRRG1 expression, along with significantly higher proportions of CD31⁺, Ly6C⁺, and F4/80⁺ cells. Notably, these trends were markedly reversed in the “OE + warfarin” group (Fig. 11A–G). These findings indicate that PRRG1 promotes enhanced tumor angiogenesis, increased monocyte recruitment, and augmented accumulation of mature tumor-associated macrophages (TAMs) within the PC tumor microenvironment, suggesting a shift toward an immunosuppressive and pro-tumorigenic state [36–38]. Notably, these PRRG1-driven immunological alterations were markedly inhibited by warfarin treatment. Collectively, these findings underscore a critical role of PRRG1 in PC tumor microenvironment.

## Discussion

PC remains one of the most aggressive and lethal malignancies, with a dismal 5-year survival rate [1]. Its insidious onset often leads to late diagnosis, and its marked resistance to therapy results in limited treatment efficacy [39, 40]. These challenges highlight the urgent need for improved early detection and more effective therapeutic strategies [41, 42]. Accordingly, identifying novel molecular targets has become a key focus in PC research to advance therapeutic development.

Pancreatic ductal adenocarcinoma (PDAC) can be broadly categorized into two major transcriptional subtypes: classical and basal-like [43, 44]. The classical subtype is characterized by well differentiated and generally shows better therapeutic responsiveness [45, 46]. In contrast, the basal-like subtype exhibits a poorly differentiated, mesenchymal-like and highly invasive phenotype, often associated with chemoresistance and poor prognosis [47, 48]. Given that PRRG1 is highly expressed in both CFPAC-1 and PATU-8988T cells, we selected these two lines for our in vitro experiments. This choice allows us to effectively capture the biological features of the two major transcriptional subtypes of PDAC—classical (CFPAC-1) and basal-like (PATU-8988T). Because classical-type subtype exhibits a higher degree of epithelial differentiation and lower intratumoral heterogeneity, it minimizes the biological noise arising from tumor-intrinsic variability. This provides a more controlled context for dissecting the regulatory mechanisms of PRRG1 and its associated signaling pathways (PI3K/Akt) in both in vitro and in vivo experiments. Moreover, the use of a Classical model offers valuable contrast to previous study [30] that mainly used basal-like models. This addresses the previous lack of a Classical subtype model. When examining the inhibitory effects of low-dose warfarin on PRRG1, we employed basal-like PDAC lines (PaTu-8988T and PANC02), which exhibit higher invasiveness and chemoresistance. This choice provides a more clinically relevant system for mimicking the aggressive and treatment-refractory nature of human PDAC.

PRRG protein family, a series of Vitamin K-dependent (VKD) single-channel integrated membrane proteins, have emerged as potential regulators of cancer progression [25, 26, 30]. Our comprehensive analysis revealed significant PRRG1 upregulation in PC. These findings align with previous reports linking PRRG1 to prognosis in malignancies [27–30]. Functional studies provided compelling evidence for PRRG1’s oncogenic role. These results were corroborated by in vivo xenograft models, where PRRG1 overexpressed markedly promoted tumor growth. Single-cell sequencing analysis further revealed preferential PRRG1 expression in PC epithelial cells, suggesting its potential involvement in driving the aggressive behavior of PC cells [49, 50]. Our study supports PRRG1 drive the progression of PC in vitro and in vivo.

Mounting evidence implicates several key signaling pathways, including MAPK/ERK [51], PI3K-Akt [52] and JAK-STAT [53], as critical drivers of oncogenesis. Our mechanistic investigations demonstrated that PRRG1 knockdown significantly reduced phosphorylation of key PI3K-Akt pathway components in PC cells in vitro. PI3K/Akt signaling pathway [54] plays a key role in the process of regulating protein synthesis, nutrient metabolism, growth factor signaling, cell growth and migration in cancer [55]. This pathway can be activated through both receptor tyrosine kinases (RTKs) [18, 56, 57] and non-receptor tyrosine kinases (NRTKs) [58, 59]. Notably, vitamin K-dependent (VKD) proteins appear to be important regulators of this pathway. For instance, GAS6, another VKD protein and shared ligand of TAM receptors (TYRO3, AXL, and MERTK), promotes bladder [60] and gastric [61] cancer progression through Akt pathway activation. Conversely, GAS6 knockdown suppresses esophageal cancer growth by inhibiting PI3K-Akt signaling [62]. Our findings position PRRG1 as a novel VKD protein that, like GAS6, promotes tumor progression through PI3K-Akt pathway activation. Although the GAS6/AXL axis has been shown to promote tumor progression through activation of the PI3K–Akt pathway, PRRG1 overexpression did not increase the expression of GAS6/AXL axis in the warfarin inhibition experiments. This suggests that PRRG1 may activates PI3K–Akt signaling through a GAS6/AXL-independent mechanism. In addition, treatment targeting PI3K/Akt signaling pathway [63] could potently inhibit PC cell growth [64]. Since LY294002 inhibited the cancer-promoting effect caused by overexpression of PRRG1, PI3K-Akt inhibitors can serve as indirect inhibitors of PRRG1.

Given the elevated expression of PRRG1 in PC, we investigated its transcriptional regulatory mechanisms. Through systematic screening and validation of potential transcription factors binding to the PRRG1 promoter, we identified Krüppel-like factor 4 (KLF4) as a key regulator. KLF4 is an evolutionarily conserved zinc finger transcription factor [65] known to play pivotal roles in tumor progression [66] and cancer stem cell maintenance [67]. Notably, KLF4 has been shown to promote colorectal cancer development by activating STAT3 signaling and inducing epithelial-mesenchymal transition (EMT) [68]. Given that clinically applicable KLF4 inhibitors are currently lacking, KLF4 suppression has mainly relied on experimental approaches using a limited number of small-molecule compounds, including kenpaullone [69] and WX2-43 [70]. In the present study, we employed KLF4 siRNA to validate that KLF4 knockdown suppresses PRRG1 expression. We speculate that therapeutic agents targeting KLF4 may serve as potential inhibitors of the KLF4–PRRG1 axis.

Warfarin, a kind of oral coumarin anticoagulant, inhibits the vitamin K oxidoreductase (VKORC1), which inducing the decrease of vitamin K hydroquinone (KH) and the inhibition of γ-glutamyl carboxylation [19, 20]. Low-dose warfarin (2uM) blocks Gas6-mediated AXL activation and inhibits PC promotion [11]. Wu et al. [30] previously proposed warfarin as a potential PRRG1 inhibitor, which reduces the protein level and downstream signaling of KRAS and EGFR elevated by PRRG1 overexpression. Our research suggested that low-dose warfarin reduces the levels of PRRG1 and Gas6/AXL axis in PC cells. In vitro and in vivo, the pro-cancer effect of PRRG1 overexpression in PC cells can be inhibited by low-dose warfarin. Multiplex IHC analysis also revealed that, warfarin reversed the PRRG1-induced alterations in the tumor immune microenvironment. These results further suggest that warfarin could be an inhibitor of PRRG1 and Gas6/AXL axis. In addition, warfarin use has been associated with improved overall survival in cancer patients [71]. For patients exhibiting high PRRG1 expression, warfarin may offer a potential targeted intervention. However, because warfarin is an anticoagulant, dose optimization and safety monitoring would be essential for future translational application. Furthermore, studies have indicated that the intracellular motifs of the PRRG family mediate protein–protein interactions [25, 30], which may serve as structurally targetable sites for future drug development.

The tumor microenvironment plays a crucial role in cancer progression through complex cellular interactions [36, 37, 50]. Of particular importance, epithelial cells contribute significantly to oncogenic processes, including epithelial-mesenchymal transition (EMT), which facilitates PC metastasis and invasion [50]. Our single-cell sequencing analysis revealed significantly elevated PRRG1 expression in PC epithelial cells compared to their normal counterparts. Macrophages and endothelial cells both participate in tumor-related inflammatory responses, driving critical pro-tumorigenic processes, such as immune evasion [36] and angiogenesis [37]. Cell-cell communication analysis demonstrated enhanced interaction between PRRG1-positive epithelial cells and these stromal components. Multiplex IHC analysis of immunocompetent models revealed that PRRG1 overexpression induced a significant increase in CD31⁺ endothelial cells, Ly6C⁺ monocytes and F4/80⁺ macrophages. Enhanced tumor angiogenesis, increased monocyte recruitment, and augmented accumulation of mature tumor-associated macrophages (TAMs) within the PC tumor microenvironment, suggesting a shift toward an immunosuppressive and pro-tumorigenic state [36–38]. Previous studies have demonstrated that anticoagulation can inhibit tumor angiogenesis [72], and inhibition of the Gas6-MerTK pathway (warfarin) reduced macrophages and neutrophils in the lungs of tumor-bearing mice [73]. Our study demonstrates that warfarin treatment reverses PRRG1-induced alterations in the tumor immune microenvironment, including enhanced angiogenesis and increased recruitment of tumor-associated macrophages (TAMs). These findings underscore a critical role of PRRG1 in the establishment of a pro-tumorigenic and immunosuppressive tumor microenvironment.

Our study identifies PRRG1, a vitamin K-dependent (VKD) single-pass transmembrane protein, as a promising therapeutic target in PC due to its critical role in promoting tumor cell proliferation and metastasis. Meanwhile, PRRG1 is involved in regulating alterations in the immune microenvironment, characterized by enhanced tumor angiogenesis, increased monocyte recruitment, and augmented accumulation of mature tumor-associated macrophages (TAMs). Distinct from previous studies, our work demonstrates that PRRG1 activates the PI3K/Akt pathway in PC and is transcriptionally regulated by KLF4. The scRNA sequencing and immunocompetent models suggested that PRRG1 facilitates the establishment of a pro-tumorigenic and immunosuppressive tumor immune microenvironment in PC, which is effectively reversed by warfarin treatment.

## Conclusion

In conclusion, our findings establish PRRG1 as a key driver of PC progression through PI3K/Akt pathway activation and KLF4-mediated transcriptional regulation. PRRG1 facilitates the establishment of a pro-tumorigenic and immunosuppressive TME in PC. Low-dose warfarin significantly suppressed the pro-tumorigenic effects the PRRG1 overexpression–driven alterations in the tumor immune microenvironment.

## Supplementary information

Figure S1

Figure S2

Figure S3

Supplementary Table

Full and uncropped western blots

Supplementary figures legends

## Data Availability

All data are available upon request.
