# Supplementary figures and images for: Identification of PRRG1 as a possible molecular target of pancreatic cancer
Source: Cell Death Dis. 2026 May 10;17(1):613. doi: 10.1038/s41419-026-08832-9 (PMC13328680; doi:10.1038/s41419-026-08832-9)

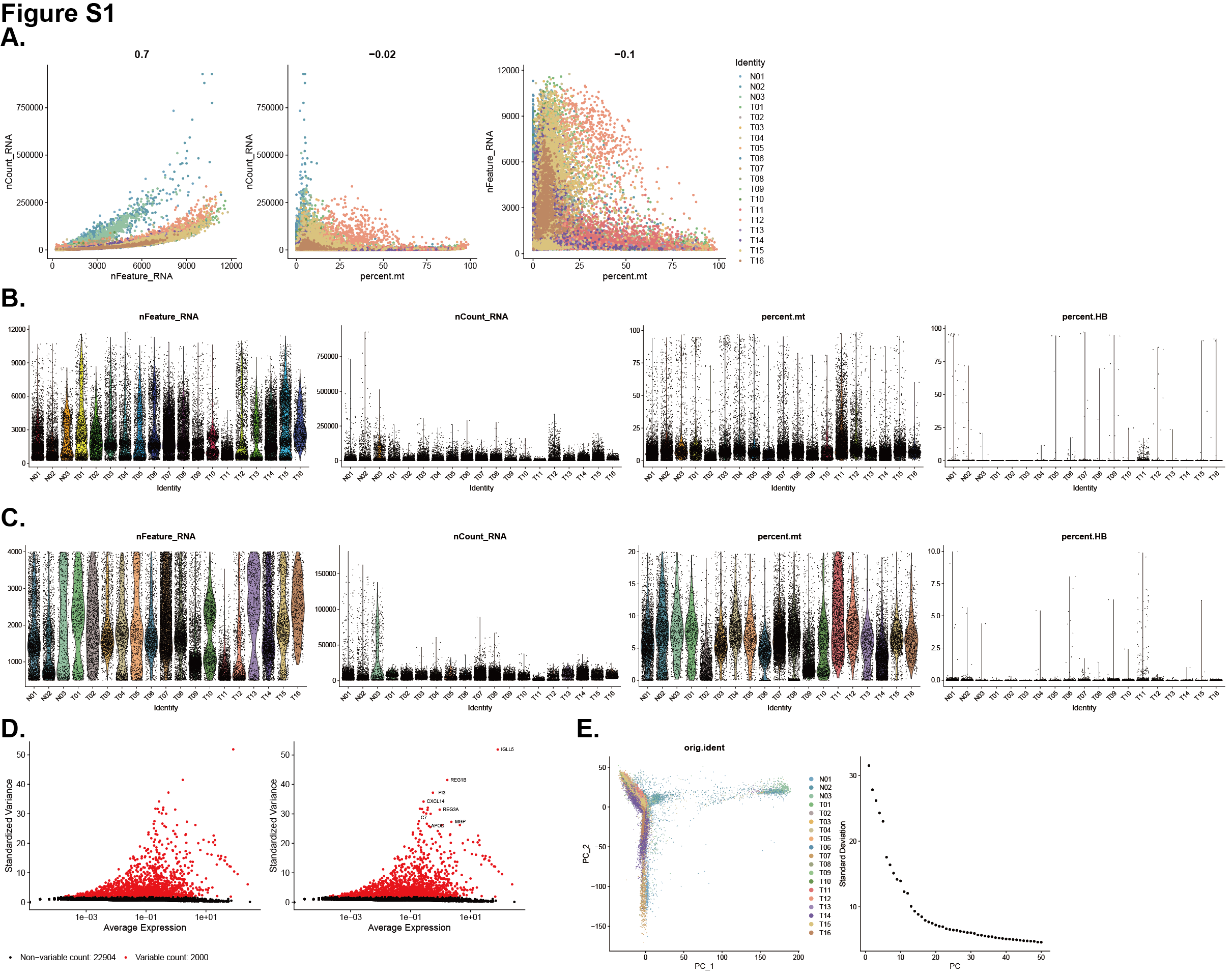

Supplement: Supplementary file 1 — Figure S1 [file 41419_2026_8832_MOESM1_ESM.png]

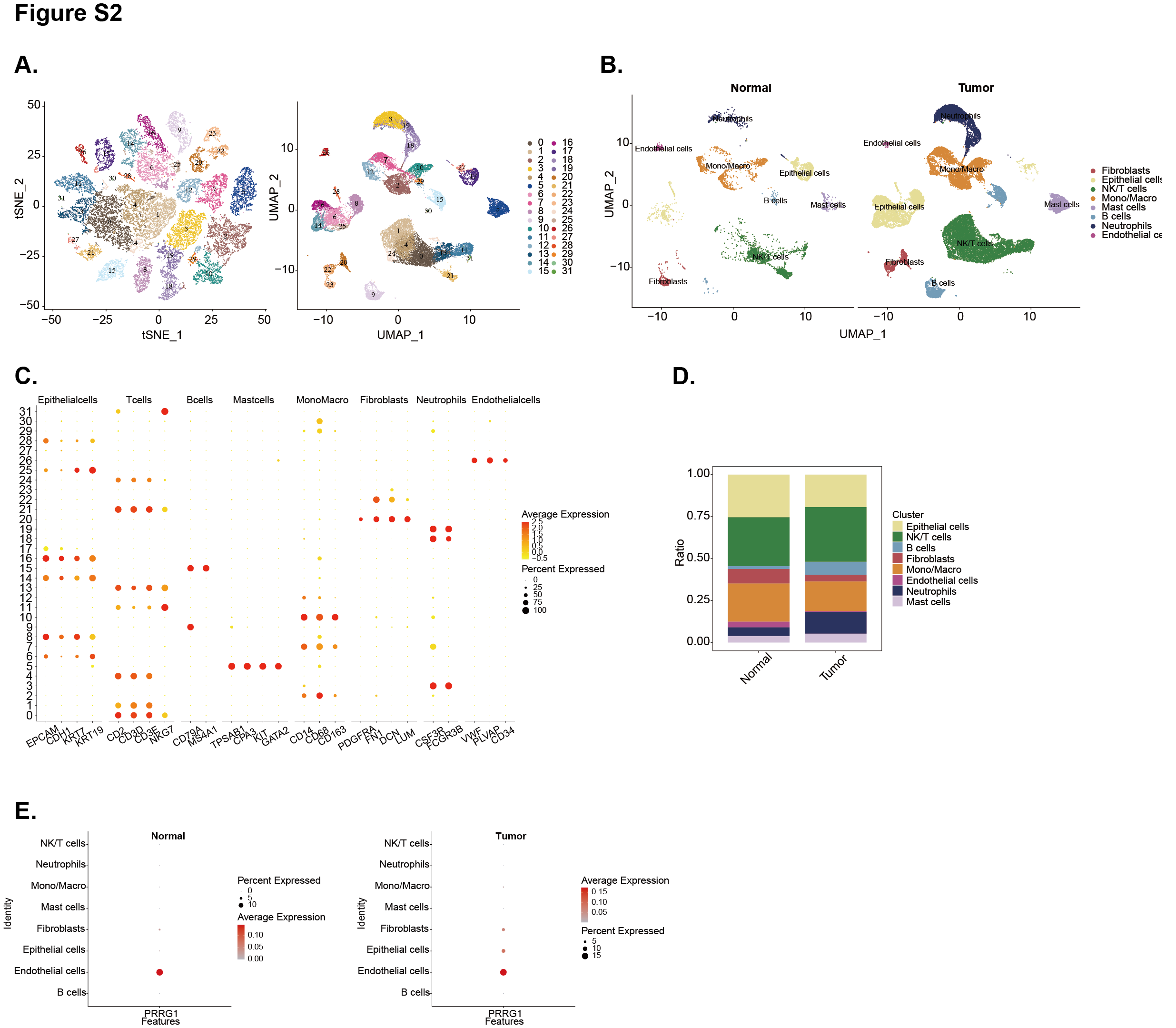

Supplement: Supplementary file 2 — Figure S2 [file 41419_2026_8832_MOESM2_ESM.png]

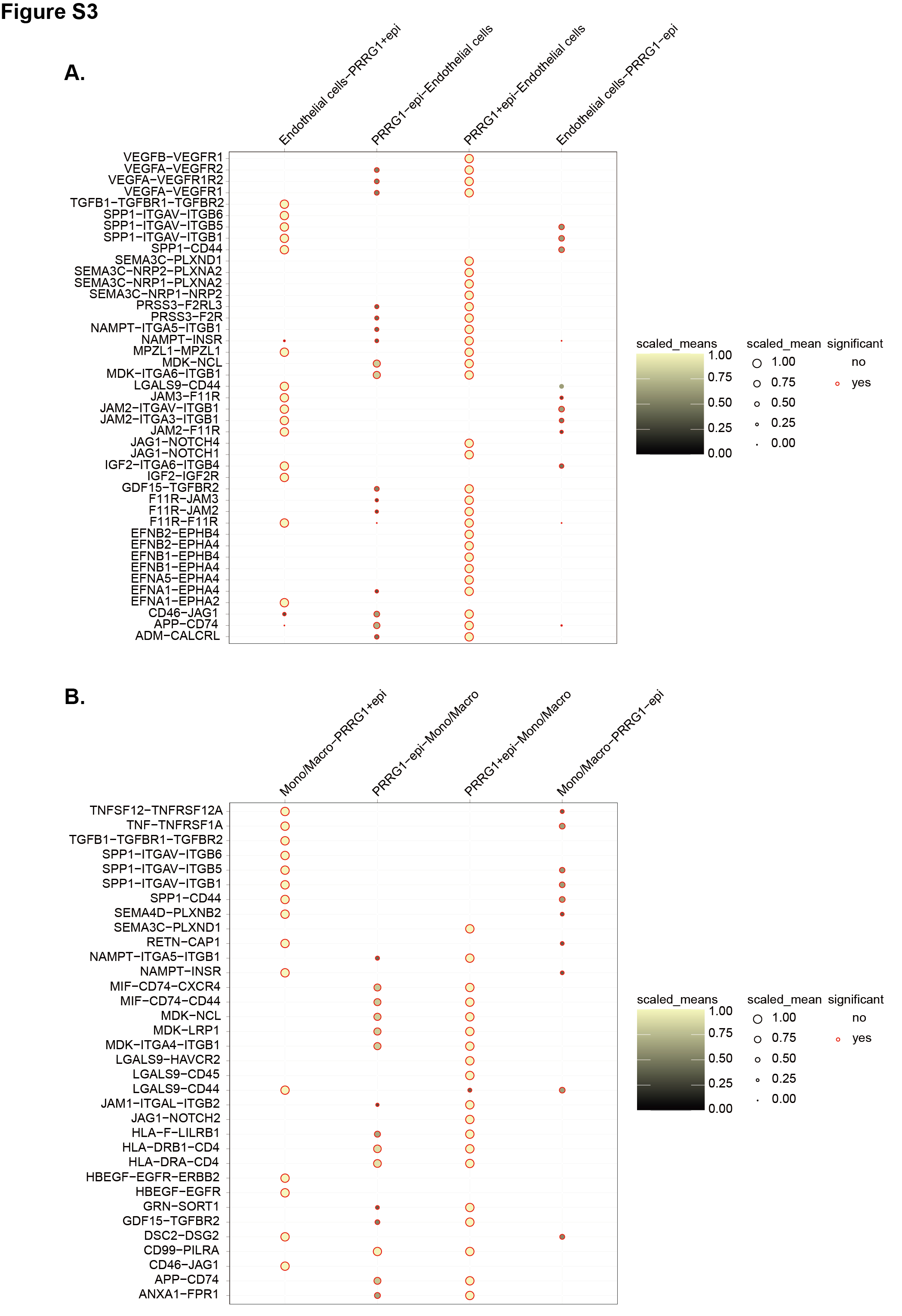

Supplement: Supplementary file 3 — Figure S3 [file 41419_2026_8832_MOESM3_ESM.png]

Figure 1

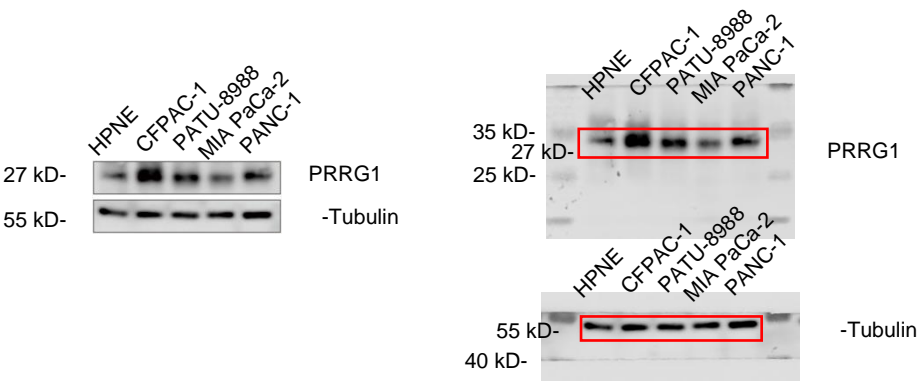

Figure 3

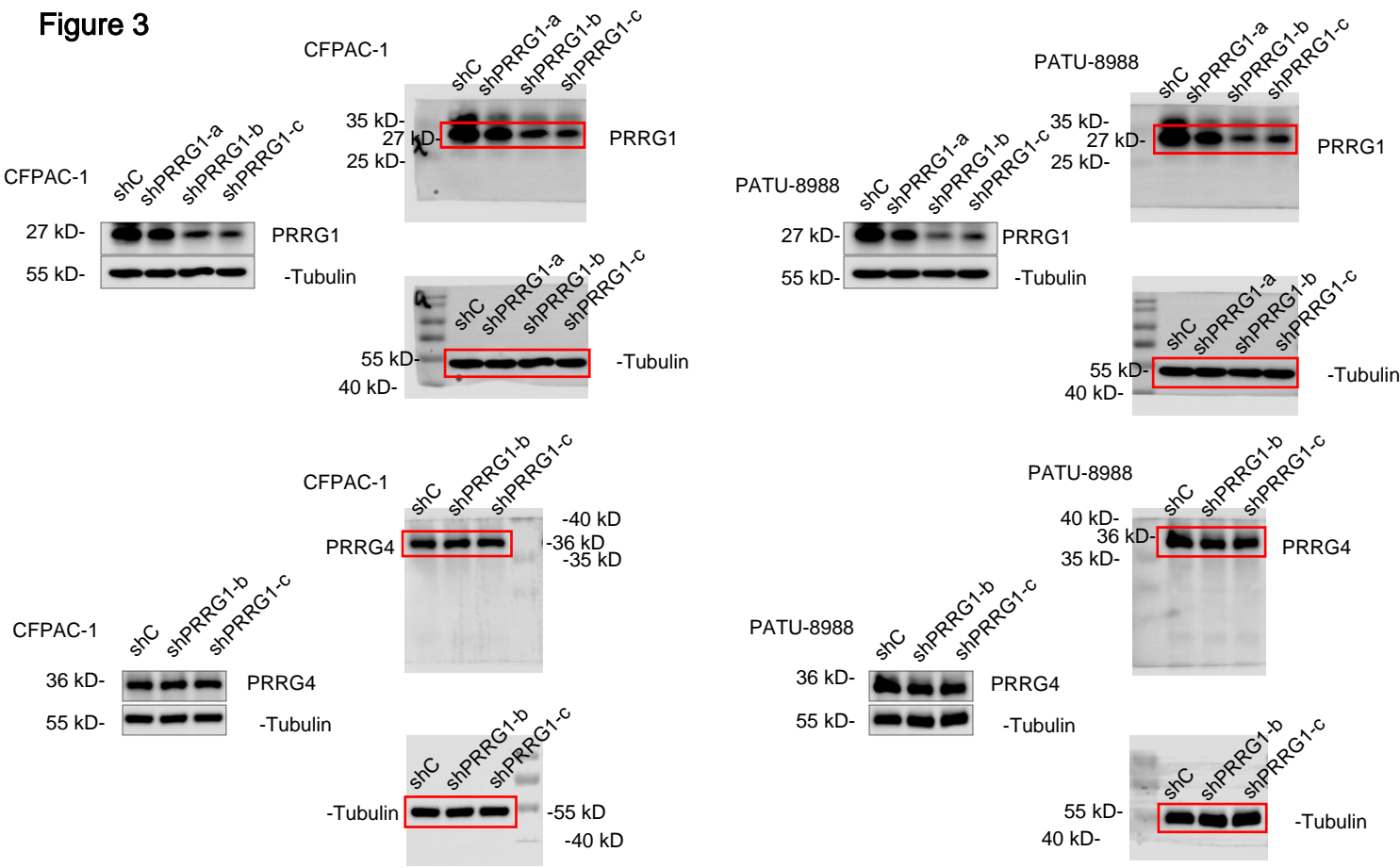

Figure 4

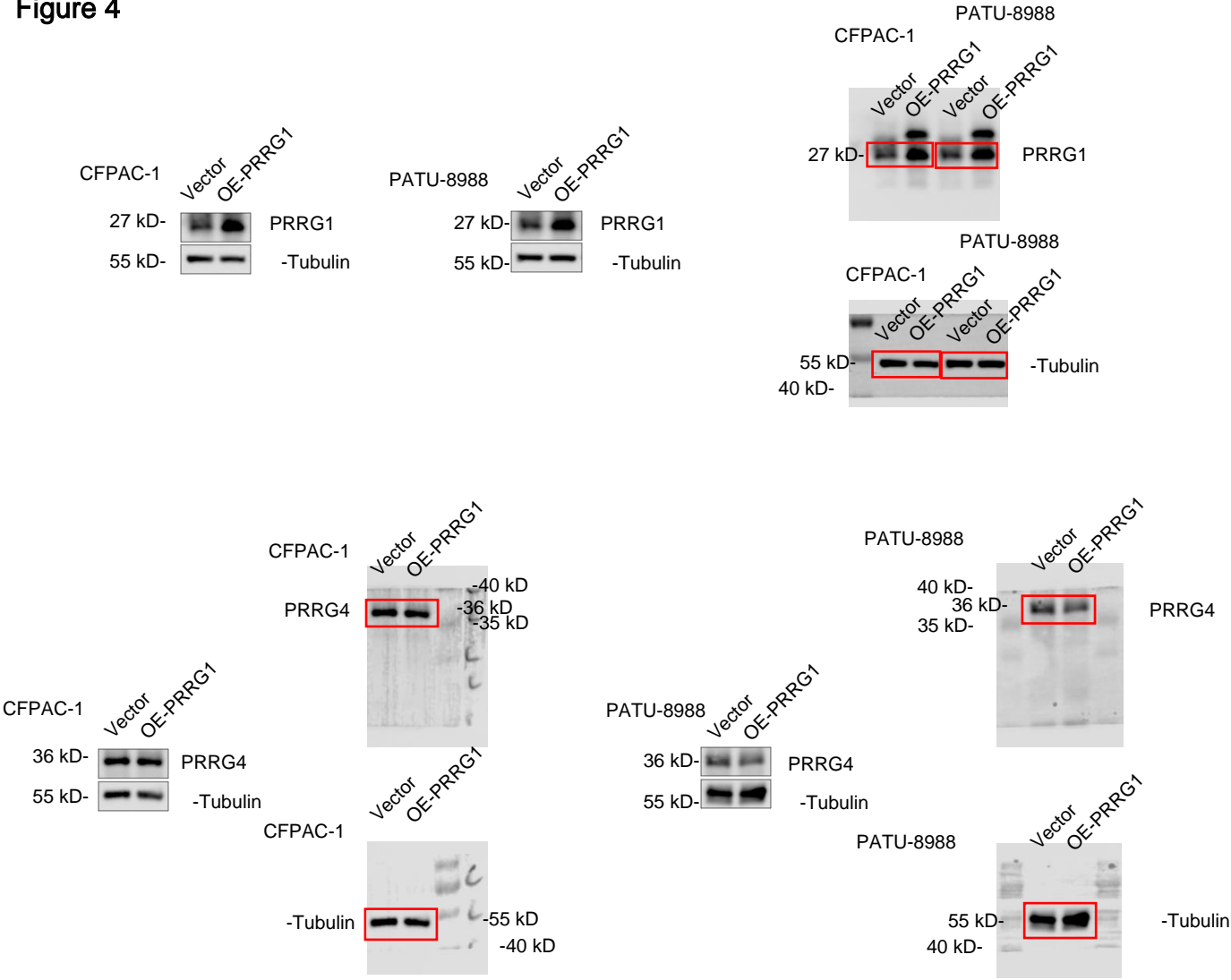

Figure 5

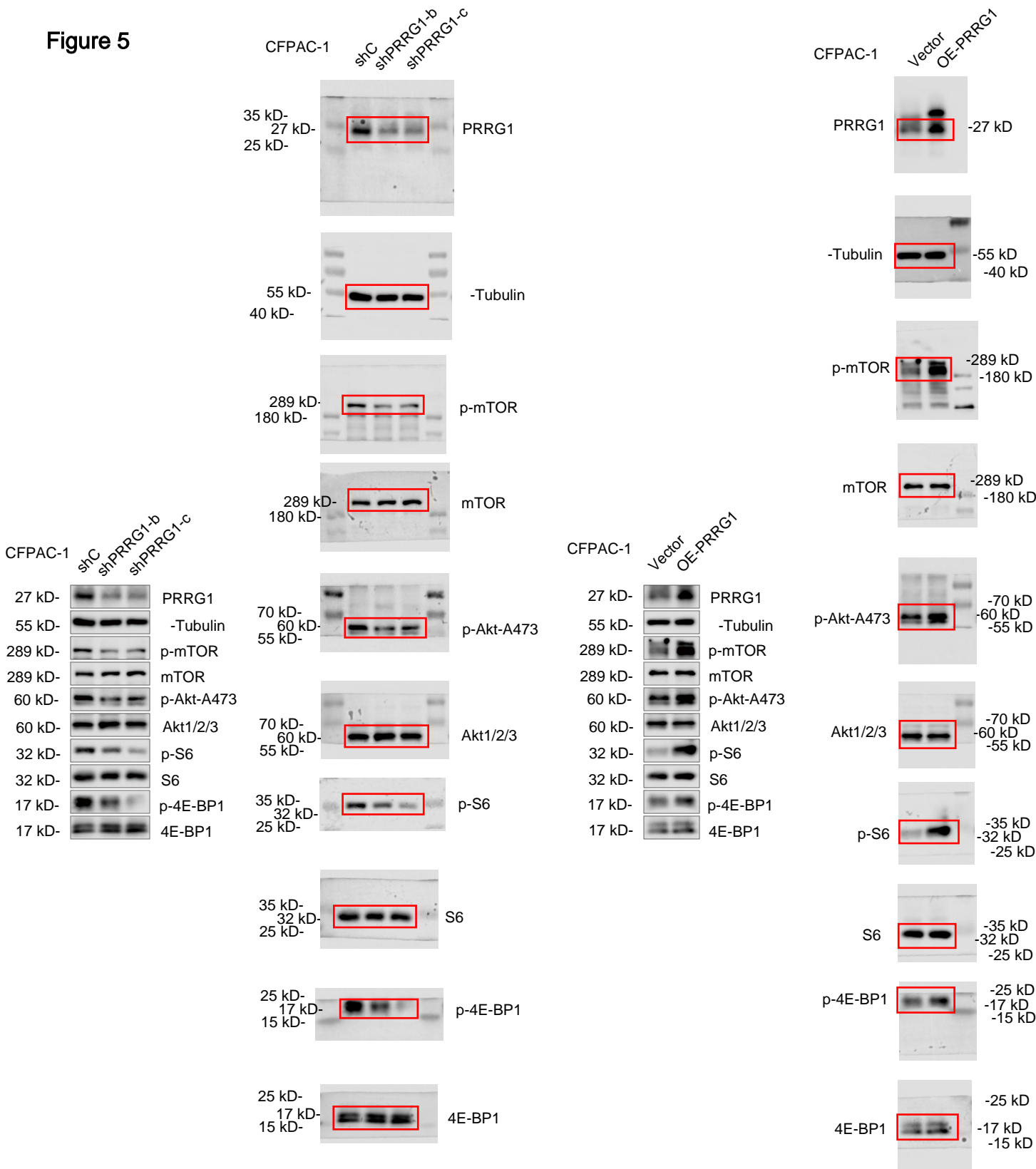

Figure 7

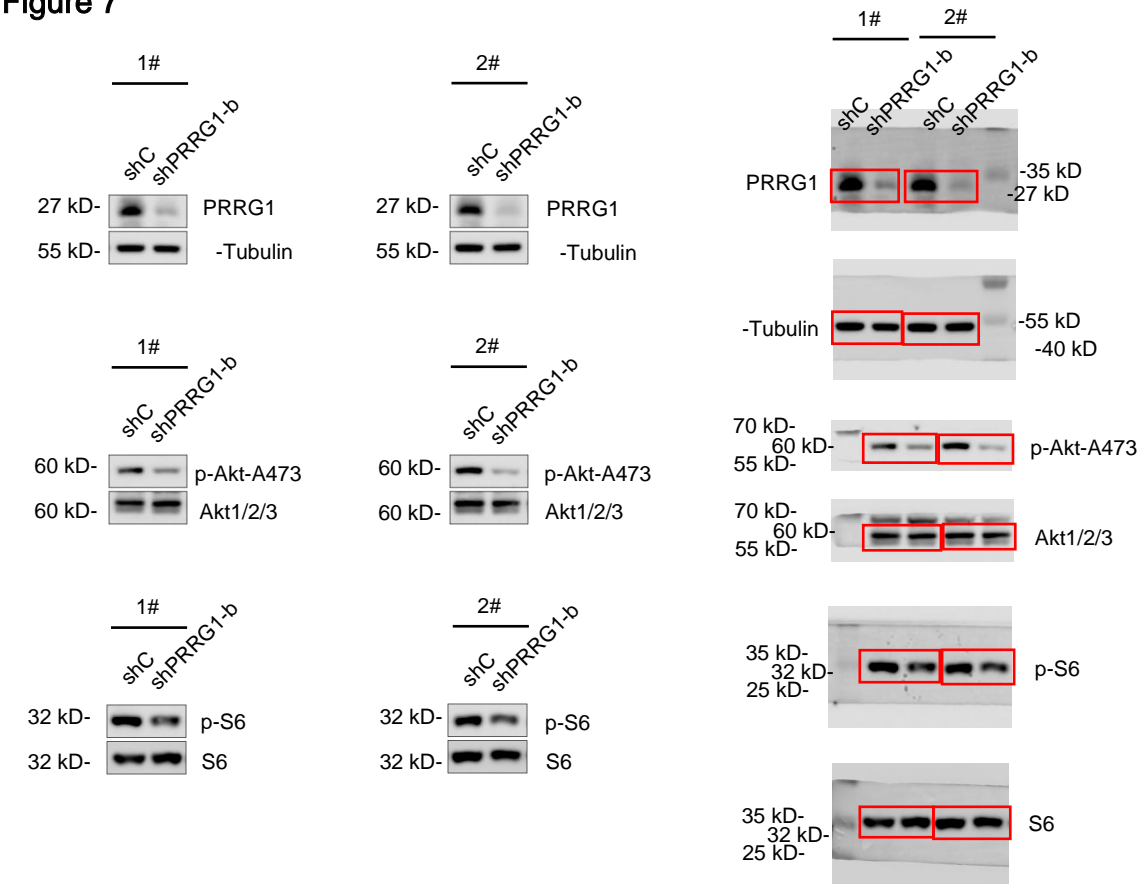

Figure 8

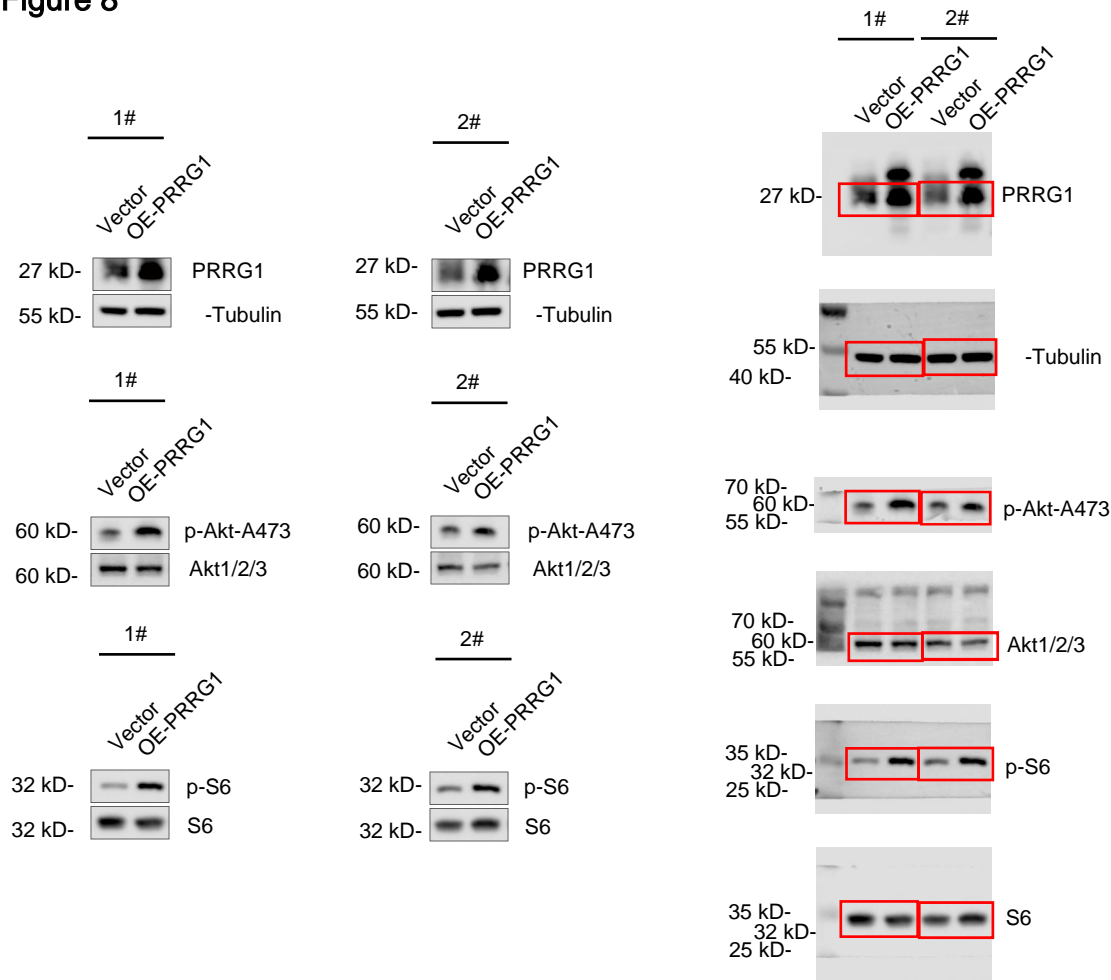

Figure 9

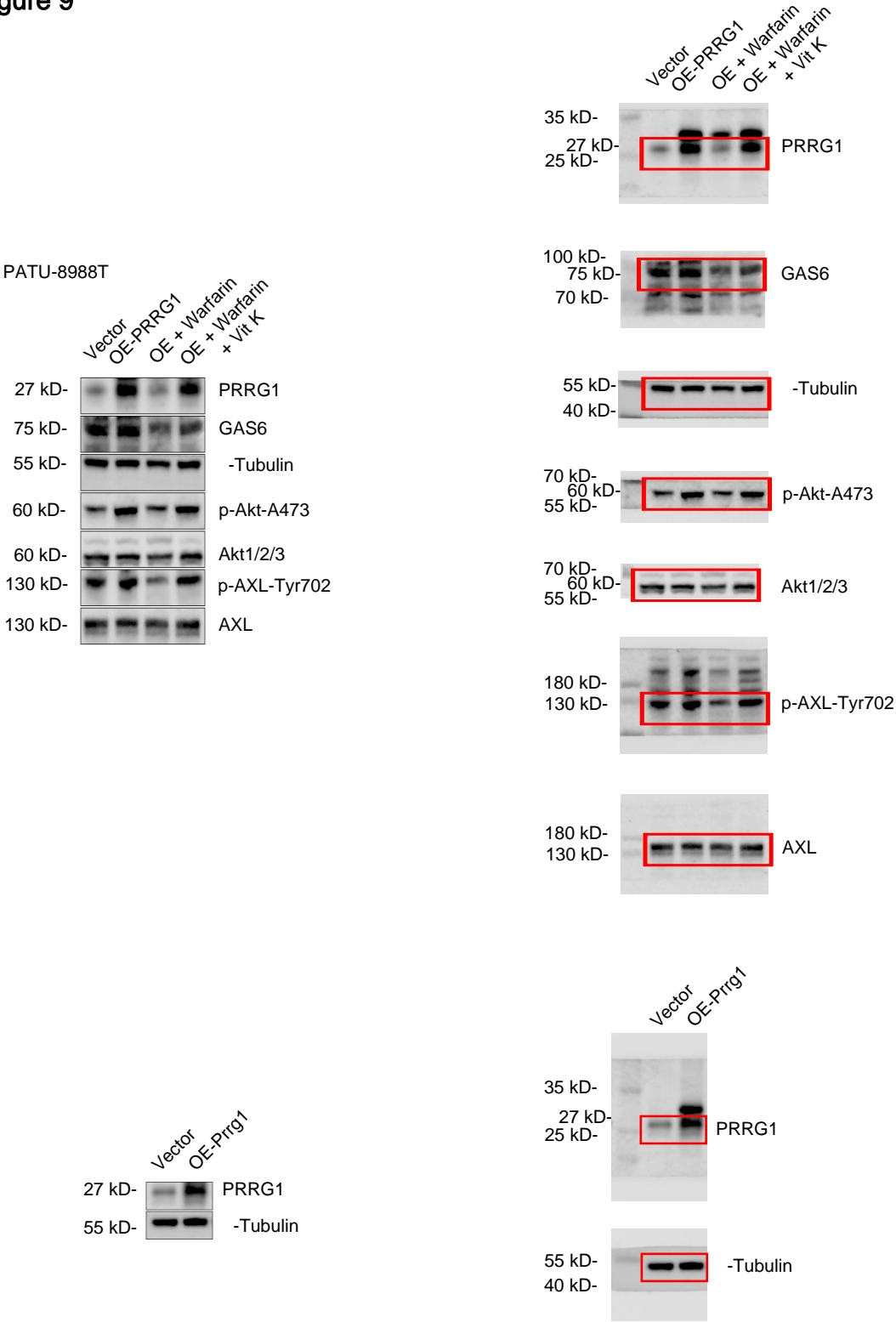

Supplement: Supplementary file 5 — Full and uncropped western blots [file 41419_2026_8832_MOESM5_ESM.pdf]
