# Supplementary material for: Identification of PRRG1 as a possible molecular target of pancreatic cancer
Source: Cell Death Dis. 2026 May 10;17(1):613. doi: 10.1038/s41419-026-08832-9 (PMC13328680; doi:10.1038/s41419-026-08832-9)
Supplement: Supplementary file 4 — Supplementary Table [file 41419_2026_8832_MOESM4_ESM.docx]

**Supplementary Table 1.** ﻿Primer sequences for PCR.

| ﻿Gene | ﻿Primer | ﻿ Sequence (5’- 3’) |
| --- | --- | --- |
| *PRRG1* | ﻿Forward Primer  ﻿Reverse Primer | TTCCTCACGGGAGAAAAAGCCAATTC  CCAGTCACTTCCTCGGTTACTCTC |
| *PRRG4* | ﻿Forward Primer  ﻿Reverse Primer | TTCACTCCCGGCAACCTAGAA  CCTGCCAAAATGCAATCGTTT |
| *KLF4* | Forward Primer  ﻿Reverse Primer | CATCTCAAGGCACACCTGCGAA  TCGGTCGCATTTTTGGCACTGG |
| *EHF* | Forward Primer  ﻿Reverse Primer | ATCAGAGGCAGTGGCTCAGCTA  ACCAGTCTTCGTCCATCCACAC |
| *PPARA* | Forward Primer  ﻿Reverse Primer | TCGGCGAGGATAGTTCTGGAAG  GACCACAGGATAAGTCACCGAG |

**Supplementary Table 2.** ﻿Lentivirus vector sequences.

| Lentivirus vector | ﻿ Sequence |
| --- | --- |
| *shPRRG1-a* | CCGGGAAATAAGACAGGGCAACATTCTCGAGAATGTTGCCCTGTCTTATTTCTTTTTG |
| *shPRRG1-b* | CCGGCAACATTGAGCGTGAGTGCAACTCGAGTTGCACTCACGCTCAATGTTGTTTTTG |
| *shPRRG1-c* | CCGGTTTGAAGAAGCAAGAGAAGCTCTCGAGAGCTTCTCTTGCTTCTTCAAATTTTTG |
| *shC* | CCGGTTCTCCGAACGTGTCACGTCTCGAGACGTGACACGTTCGGAGAATTTTTG |

**Supplementary Table 3.** ﻿siRNA sequences.

| ﻿siRNA | ﻿Fragment | ﻿ Sequence (5’- 3’) |
| --- | --- | --- |
| *siKLF4* | ﻿Sense RNA  ﻿Antisense RNA | GAGAGACCGAGGAGUUCAATT  UUGAACUCCUCGGUCUCUCTT |
| *siEHF* | ﻿Sense RNA  ﻿Antisense RNA | CCACACACAAUGUCAUUGUTT  ACAAUGACAUUGUGUGUGGTT |
| *siPPARA* | ﻿Sense RNA  ﻿Antisense RNA | GGAGCAUUGAACAUCGAAUTT  AUUCGAUGUUCAAUGCUCCTT |
| *siC* | Sense RNA  ﻿Antisense RNA | UUCUCCGAACGUGUCACGUTT  ACGUGACACGUUCGGAGAATT |
