# Supplementary material for: Identification of PRRG1 as a possible molecular target of pancreatic cancer
Source: Cell Death Dis. 2026 May 10;17(1):613. doi: 10.1038/s41419-026-08832-9 (PMC13328680; doi:10.1038/s41419-026-08832-9)
Supplement: Supplementary file 6 — Supplementary figures legends [file 41419_2026_8832_MOESM6_ESM.docx]

**Figure S1. Data quality control and filtration**

Correlation between UMI counts with mRNA levels, and correlation between mitochon-drial gene expression with UMI counts or mRNA levels were shown (**A**).

We filtered cells not meeting quality standards after further examination, shown by scatter diagram (**B** and **C**). 2000 highly variable genes were shown by volcano map (**D**). Principal components identified by PCA were demonstrated by scatter diagram (**E**).

**Figure S2.** **Cell clustering and annotation**

The t-SNE and UMAP algorithms classified the cells into final 32 clusters (**A**). Dot plot showing the expression pattern of epithelial cells, macrophages, T cells, endothelial cells and so on (**B**). Different cellular subsets in normal and tumor samples were demonstrated by UMAP (**C**) and the proportion were shown in stacked bar charts (**D**). PRRG1 expression of different cellular subsets in normal and tumor samples were demonstrated by bubble chart (**E**).

**Figure S3. Cell–cell communication analysis**

Cell chat between PRRG1- positive / negative group with Mono/Macro cells (**A**) or endothelial cells (**B**) were shown by bubble chart.
